# A randomized trial on the effect of transcutaneous electrical nerve stimulator on glycemic control in patients with type 2 diabetes

**DOI:** 10.1038/s41598-023-29791-7

**Published:** 2023-02-15

**Authors:** Jin-Ying Lu, Horng-Yih Ou, Chung-Ze Wu, Chwen-Yi Yang, Ju-Ying Jiang, Chieh-Hsiang Lu, Yi-Der Jiang, Tien-Jyun Chang, Yi-Cheng Chang, Meng-Lun Hsieh, Wan-Chen Wu, Hung-Yuan Li, Ye-Fong Du, Ching-Han Lin, Hao-Chang Hung, Kai-Jen Tien, Nai-Cheng Yeh, Shang-Yu Lee, Hui-I. Yu, Lee-Ming Chuang

**Affiliations:** 1 Division of Endocrinology and Metabolism, Department of Internal Medicine, National Taiwan University Hospital, Taipei, Taiwan; 2 Department of Laboratory Medicine, National Taiwan University Hospital, Taipei, Taiwan; 3 Division of Endocrinology and Metabolism, Department of Internal Medicine, National Cheng Kung University Hospital, Tainan, Taiwan; 4 College of Medicine, National Cheng Kung University, Tainan, Taiwan; 5 Division of Endocrinology and Metabolism, Department of Internal Medicine, School of Medicine, College of Medicine, Taipei Medical University, Taipei, Taiwan; 6 Division of Endocrinology and Metabolism, Department of Internal Medicine, Shuang Ho Hospital, Taipei Medical University, Taipei, Taiwan; 7 Division of Endocrinology and Metabolism, Department of Internal Medicine, Chi Mei Medical Center, Tainan, Taiwan; 8 Division of Endocrinology and Metabolism, Department of Internal Medicine, Far Eastern Memorial Hospital, New Taipei City, Taiwan; 9 Division of Endocrinology and Metabolism, Department of Internal Medicine, Ditmanson Chia-Yi Christian Hospital, Chia-Yi City, Taiwan; 10 Lutheran Medical Foundation, Kaohsiung Christian Hospital, Kaohsiung, Taiwan; 11 Graduate Institute of Medical Genomics and Proteomics, National Taiwan University, Taipei, Taiwan; 12 Institute of Biomedical Sciences, Academia Sinica, Taipei, Taiwan; 13 Institute of Epidemiology and Preventive Medicine, National Taiwan University, Taipei, Taiwan; 14 Present Address: Graduate Institute of Clinical Medicine, Medical College, National Taiwan University, No.1, Sec.1, Ren-Ai Rd., Taipei, 10051 Taiwan

**Keywords:** Biotechnology, Diseases, Endocrinology, Medical research

## Abstract

Transcutaneous electrical nerve stimulator (TENS) has been demonstrated to be beneficial in glycemic control in animal models, but its application in humans has not been well studied. We randomly assigned 160 patients with type 2 diabetes on oral antidiabetic drugs 1:1 to the TENS study device (n = 81) and placebo (n = 79). 147 (92%) randomized participants (mean [SD] age 59 [10] years, 92 men [58%], mean [SD] baseline HbA_1c_ level 8.1% [0.6%]) completed the trial. At week 20, HbA_1c_ decreased from 8.1% to 7.9% in the TENS group (− 0.2% [95% CI − 0.4% to − 0.1%]) and from 8.1% to 7.8% in the placebo group (− 0.3% [95% CI − 0.5% to − 0.2%]) (*P* = 0.821). Glycemic variability, measured as mean amplitude of glycemic excursion (MAGE) at week 20 were significantly different in the TENS group vs. the placebo group (66 mg/dL [95% CI 58, 73] vs. 79 mg/dL [95% CI 72, 87]) (*P* = 0.009). Our study provides the clinical evidence for the first time in humans that TENS does not demonstrate a statistically significant HbA_1c_ reduction. However, it is a safe complementary therapy to improve MAGE in patients with type 2 diabetes.

## Introduction

Despite numerous treatment options available, many patients with type 2 diabetes fail to achieve glycemic targets, leading to a significantly increased risk for diabetic chronic complications and related morbidity/mortality^1^. There is an urgent need to develop simple alternative or complementary therapeutic strategies for the treatment of type 2 diabetes^2^.

Transcutaneous electrical nerve stimulator (TENS) is an approach that applies electrical impulses generated by specifically designed devices, and delivered through electrodes placed on the skin^3^. Scientific evidence suggest that TENS represents an effective tool to treat numerous conditions including pain relief, modulation of autonomic nervous system, and control of diabetes^4–6^. In rat models, vagus nerve stimulation has been shown to modulate glycemia by affecting glucagon and insulin secretions, and high-frequency 40 kHz stimulation might potentially be applied to the treatment of type 2 diabetes^7^. Besides, a novel physiological effect of magnetostatic and electrostatic fields has been demonstrated for the long-term, noninvasive management of type 2 diabetes in mouse models^8^. Recently, a study in STZ-induced diabetes mellitus in mouse models showed that microcurrent electrical nerve stimulation exhibited effects similar to anti-diabetic drugs, through the peroxisome proliferator-activated receptor (PPAR), bile secretion, and insulin signaling pathways^9^. In a pre-clinical setting, we have proven that our TENS device significantly improved glucose parameters in a diabetic mouse model^10^, as indicated by fasting plasma glucose (FPG) and glycosylated hemoglobin (HbA_1c_) (Appendix in Supplementary Material). Besides, TENS intervention reversed the pathological changes in liver, kidney, and pancreas in mice with diabetes mellitus induced by streptozotocin. The results indicated that TENS could reduce FPG and HbA_1c_ and was safe in animal models.

However, current data in humans are scarce. The effect of TENS has been proved by a new exercise method for treating postprandial hyperglycemia in 11 patients with type 2 diabetes^11^, and low-frequency neuromuscular electrical stimulation has been reported with significant positive correlation between the intensity of stimulation and changes in blood glucose in 8 participants with type 2 diabetes^12^. The role of TENS in the treatment of type 2 diabetes of human beings remains to be elucidated. Accordingly, in this phase 3 clinical study, we aim to determine the clinical efficacy of TENS by measuring glycemic parameters including HbA_1c_, FPG, and body weight at week 0, 2, 4, 8, 12, 16, 20, and 7-point self-monitoring of blood glucose (SMBG) at week 0, 4, 12, 20 in the study period. We performed the study on patients with type 2 diabetes treated with OADs to avoid the complexity and heterogeneity caused by injectables. Mean amplitude of glycemic excursion (MAGE) was calculated using the 7-point SMBG data to represent an indicator of glycemic variability (GV). We also evaluate the safety profile of TENS, including records of any treatment-emergent adverse events (TEAEs), vital signs, rescue medication(s) for hyperglycemia, and hypoglycemic events.

## Results

### Baseline demographics and clinical characteristics

A total of 207 subjects were screened, and 160 subjects fulfilling the eligibility criteria were enrolled and randomized and they comprised the safety population. Of these, 155 subjects were included in ITT population, while 147 subjects were included in PP population. Six subjects withdrew early from this study and did not complete the 20 weeks of treatment. Two participants violated exclusion criteria and another two participants withdrew the consent. The primary reasons for withdrawal are summarized in Supplementary Table S1. Subject disposition flowchart of each treatment group is displayed in Supplementary Fig. S2. The overall drop-out rates are 8.6% in the TENS group and 7.5% in the placebo group (both < 10%).

The demographic and clinical characteristics at baseline are summarized in Table 1 for ITT population and in Supplementary Table S2 for PP population and were well balanced between the TENS and the placebo groups. In ITT population, the mean ages were 60 (10) in the TENS group and 59 (9) years in the placebo group. More than half of the subjects were men (54% in the TENS group and 61% in the placebo group). FPG levels were higher in the TENS group when compared to the placebo group (168 (51) vs. 156 (39) mg/dL, *P* = 0.086). More patients in the TENS group used SU when compared to placebo (9/78 vs. 3/77, *P* = 0.130). The demographic characteristics of PP was like those of ITT population.

**Table 1:** Baseline Demographics, Clinical Characteristics and Prescribed Oral Antidiabetic Drugs.

	No. (%)	Placebo (n = 77)	*P* value
	TENS (n = 78)		
Age, mean (SD), y	59 (10)	59 (9)	0.615
Sex			0.365
Female	36 (46)	30 (39)	
Male	42 (54)	47 (61)	
Height, mean (SD), cm	163 (10)	164 (8)	0.574
Weight, mean (SD), kg	72 (16)	73 (14)	0.515
BMI, mean (SD), kg/m^2^	27 (4)	27 (4)	0.594
HbA_1c_, mean (SD), %	8.1 (0.5)	8.1 (0.5)	0.989
FPG, mean (SD), mg/dL	168 (50)	155 (39)	0.086
Mean 7-point SMBG, mean (SD), mg/dL	184 (33)	180 (28)	0.746
MAGE, mean (SD), mg/dL	85 (33)	88 (30)	0.354
SBP, mean (SD), mmHg	128 (15)	127 (13)	0.746
DBP, mean (SD), mmHg	77 (10)	78 (9)	0.611
TC, mean (SD), mg/dL	160 (31)	162 (31)	0.588
TG, mean (SD), mg/dL	158 (94)	154 (95)	0.863
LDL-C, mean (SD), mg/dL	90 (24)	93 (23)	0.230
HDL-C, mean (SD), mg/dL	46 (13)	46 (12)	0.790
OADs prescribed			
Metformin	73 (94)	75 (97)	0.442*
SU	9 (12)	3 (4)	0.130*
DPP4i	24 (31)	25 (33)	0.956
SGLT2i	16 (21)	24 (31)	0.183
TZD	17 (22)	13 (17)	0.568
AGi	7 (9)	6 (8)	1.000

Data are presented in means ± SD unless otherwise indicated. Two-sample *t*-test was conducted for continuous variable; Chi-square test or Fisher’s exact test were conducted for categorical variable.

*TENS* the study device “Dragon Waves Resonant Home Care” Electronic Nerve Stimulator, *BMI* body mass index, *HbA*_*1c*_ glycosylated hemoglobin, *FPG* fasting plasma glucose, *SMBG* self-monitoring blood glucose, *MAGE* mean amplitude of glycemic excursion, *SBP* systolic blood pressure, *DBP* diastolic blood pressure, *TC* total cholesterol; *TG* triglyceride, *LDL-C* low-density lipoprotein cholesterol, *HDL-C* high-density lipoprotein cholesterol, *OADs* oral anti-hyperglycemic drugs, *SU* sulfonylurea, *DPP4i* dipeptidyl peptidase 4 inhibitor; *SGLT2i* sodium-glucose cotransporter 2 inhibitor, *TZD* thiazolidinedione, *AGi* α-glucosidase inhibitor.

Study treatment compliance in ITT and PP populations are summarized in Supplementary Table S3. The study expected subjects to use the study device 60 min per day, at least 5 days per week for consecutive 20 weeks (overall 6000 min). All treatments administration were recorded by each study subject, and the treatment compliance rate achieved over 100% in both groups.

### Primary outcome

The primary efficacy endpoint was HbA_1c_ at 20 weeks of treatment vs. baseline, shown in Table 2 and Supplementary Table S4. HbA_1c_ at each visit for ITT and PP populations is shown in Supplementary Table S5. In general, HbA_1c_ gradually decreased from week 0 to week 12 and remained stable afterwards in both the TENS and the placebo groups. The primary endpoint analysis of HbA_1c_ at week 20 did not demonstrate a statistically significant difference between the TENS and the placebo groups (*P* = 0.540 in ITT, *P* = 0.679 in PP population). In ITT population, HbA_1c_ decreased 0.24 (0.64)% in the TENS group and 0.33 (0.82)% in the placebo group at week 20 when compared to baseline. In PP population, HbA_1c_ decreased 0.27 (0.66)% in the TENS group and 0.34 (0.84)% in the placebo group at week 20 when compared to baseline (Table 2, Supplementary Table S4).

**Table 2:** Glycemic Outcomes.

	Mean (SD)				*P* value
	Baseline		20 weeks		
	TENS	Placebo	TENS	Placebo	
Primary outcome					
No	78	77	77	73	
HbA_1c_, %,	8.1 (0.5)	8.1 (0.5)	7.9 (0.8)	7.8 (0.8)	
Change from baseline, %, [95% CI]			-0.2 (0.6) [− 0.4, − 0.1]	− 0.3 (0.8) [− 0.5, − 0.2]	0.821
Secondary outcomes					
No	78	77	77	73	
HbA_1c_ < 7%, No. (%)	0	0	8 (10)	8 (11)	0.910
No	78	77	77	73	
FPG, mg/dL	168 (50)	155 (39)	157 (45)	151 (42)	
Change from baseline, mg/dL, [95% CI]			− 11 (45) [− 22, − 1]	− 4 (56) [− 16, 9]	0.369
No	78	76	77	73	
Mean 7-point SMBG	184 (33)	180 (28)	173 (34)	172 (34)	
Change from baseline, mg/dL, [95% CI]			− 11 (34) [− 19, − 3]	− 8 (36) [− 17, 0]	0.730
Additional outcome					
No	78	76	77	73	
MAGE, mg/dL	85 (33)	88 (30)	66 (33)	79 (33)	
Change from baseline, mg/dL, [95% CI]			− 19 (39) [− 28, − 10]	− 9 (43) [− 19, 2]	0.087
Exploratory outcomes					
No	78	77	77	73	
CRP, mg/dL	0.22 (0.30)	0.24 (0.07)	0.16 (0.23)	0.38 (1.12)	
Change from baseline, mg/dL, [95% CI]			− 0.06 (− 0.02) [− 0.13, 0.01]	0.13 (− 0.003) [− 0.14, 0.40]	0.181
No	78	77	77	73	
FGF-21, ng/mL	0.29 (0.25)	0.35 (0.33)	0.24 (0.17)	0.32 (0.30)	
Change from baseline, ng/mL, [95% CI]			− 0.05 (− 0.01) [− 0.09, 0.00]	− 0.03 (− 0.01) [− 0.08, 0.02]	0.789

Two-sample *t*-test (continuous variable) or Chi-square Test (categorical variable) was conducted. If normal assumption was violated, Wilcoxon rank sum test was applied.

*SD* standard deviation, *CI* confidence interval, *HbA*_*1c*_ glycosylated hemoglobin, *FPG* fasting plasma glucose, *SMBG* self-monitoring blood glucose, *MAGE* mean amplitude of glycemic excursion, *CRP* C-reactive protein, *FGF-21* fibroblast growth factor-21.

### Secondary outcome

The proportion of subjects achieving HbA_1c_ of < 7% at each visit for ITT and PP populations are shown in Supplementary Table S6. The proportion of subjects who achieved HbA_1c_ of < 7% gradually increased since week 4 in both groups. In ITT population, there were 10.4% of subjects in the TENS group and 11% of subjects in placebo group achieved HbA_1c_ of < 7% at week 20. The proportion of subjects achieving HbA_1c_ of < 7% in both groups in PP population was like that in ITT population.

FPG at each visit for ITT and PP populations are shown in Supplementary Table S7. FPG gradually decreased since week 0 in both groups. No statistically significant difference between groups was observed. In ITT population, FPG decreased 11 (45) mg/dL in the TENS group and 4 (56) mg/dL in the placebo group at week 20 compared to baseline. In PP population, FPG decreased 12 (46) mg/dL in the TENS group and 4 (57) mg/dL in the placebo group at week 20 compared to baseline.

According to subgroup analysis (Supplementary Table S8), female in the TENS but not the placebo group exhibited a statistically significant HbA_1c_ reduction from baseline in ITT (*P* = 0.0320) and PP population (*P* = 0.0049). HbA_1c_ and FPG from baseline to week 20 significantly decreased in the TENS subgroup of subjects with body mass index (BMI) ≥ 26.9 kg/m^2^ (the median) in both ITT and PP populations (HbA_1c_ in ITT: *P* = 0.0309, PP: *P* = 0.0218; FPG in ITT: *P* = 0.0425; PP: *P* = 0.0325). The change was not statistically significant in the corresponding placebo subgroup. Subjects having DM duration for more than 9 years in the TENS group also displayed a significant HbA_1c_ reduction from baseline to week 20 in both populations (ITT: *P* = 0.0282; PP: *P* = 0.0282); again, the change was not statistically significant in the corresponding placebo subgroup.

### Other outcomes

**Figure 1:**
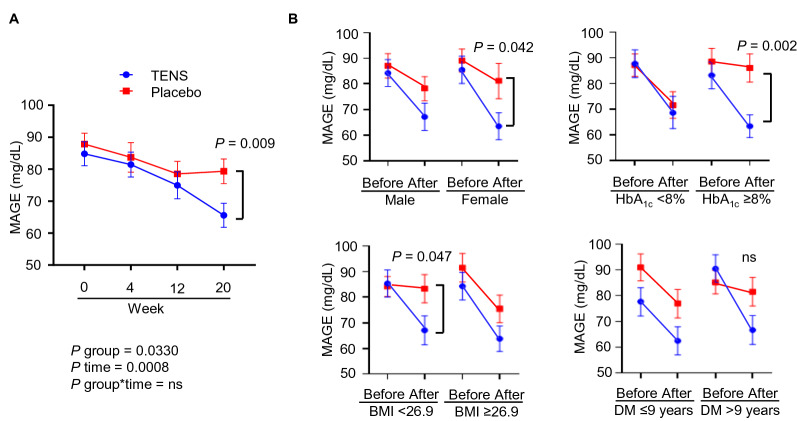
(**A**) Mean amplitude of glycemic excursion (MAGE) from week 0 to week 20 in the Transcutaneous Electrical Nerve Stimulator (TENS) vs. the placebo group. (**B**) Subgroup analysis of MAGE before and after treatment by gender, HbA_1c_ (%), BMI (kg/m^2^) and duration of DM. Mixed model two–way ANOVA and two-sample *t*-test were carried out and presented with means ± standard error of mean (SEM), ns, non-significant.

The 7-point SMBG was measured immediately before breakfast, lunch, and dinner, two hours after each meal, and at bedtime, at week 0, 4, 12 and 20. In the TENS group, the 7-point SMBG maintained relatively more stable GV compared to the placebo group, as reflected by the significant difference of MAGE at week 20 between the TENS group and the placebo (66 (33) mg/dL [95% CI 58, 73] vs. 79 (33) mg/dL [95% CI 72, 87]) (Table 2, Supplementary Table S10, and Fig. 1A) (two-sample *t*-test showed a significant post-treatment difference with *P* = 0.009; mixed model 2-way ANOVA showed a significant group effect with *P* = 0.033 and a significant time effect with *P* = 0.0008). Similar trends of greater MAGE decline were observed in the TENS group as compared to the placebo group in both genders, in patients with either higher or lower HbA_1c_ and BMI, and in patients with either longer or shorter duration of DM (Fig. 1B). Statistically significant differences of MAGE after treatment in the TENS group vs. the placebo group were noted in female (*P* = 0.042), in patients with HbA_1c_ ≥ 8.0% (*P* = 0.002), in patients with BMI < 26.9 kg/m^2^ (*P* = 0.047).

Following 20 weeks of treatment, most subjects in the TENS and the placebo groups maintained stable OAD regimens in the maintenance period of the study. Overall, four subjects had OAD regimen change: two in the TENS group, and two in the placebo group (Supplementary Table S11). The vital signs including blood pressure, pulse, respiratory rate, and body temperature in both treatment groups remained stable over the study period with no significant difference from baseline (Supplementary Table S12). Comparisons of lipid profile between groups are shown in Supplementary Table S13. Overall, total cholesterol (TC) and triglyceride (TG) showed significant decline in the placebo group, but high-density lipoprotein cholesterol (HDL-C) showed significant elevation in the TENS group.

**Figure 2:**
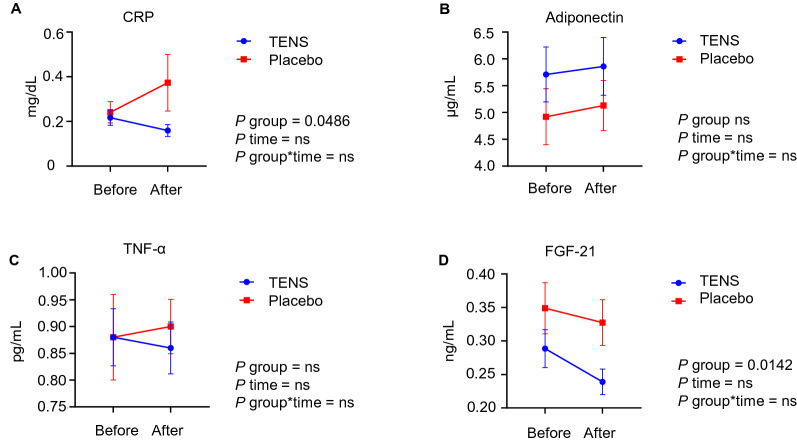
Change of exploratory parameters from before (week 0) and after (week 20) treatment in Transcutaneous Electrical Nerve Stimulator (TENS) vs. the placebo group. Statistical analysis with mixed model two–way ANOVA was carried out. Values are means ± standard error of mean (SEM), ns, non-significant. CRP, C-reactive protein; TNF-α, tumor necrosis factor-α; FGF-21, fibroblast growth factor-21.

Exploratory biomarkers including CRP, adiponectin, TNF-α, and FGF-21 were performed at week 0 and 20. Figure 2, Table 2, and Supplementary Table S14 summarize the levels and mean change from week 0 to 20 in exploratory laboratory parameters. CRP decreased 0.06 (0.02) in the TENS group (*P* = 0.022) and increased 0.13 (0.003) mg/dL (*P* = 0.848) in the placebo group from week 0 to week 20 (two sample *t*-test *P* = 0.095; mixed model 2-way ANOVA showed a significant group effect with *P* = 0.0486). TNF-α decreased 0.03 (0.45) pg/mL in the TENS group and increased 0.03 (0.55) pg/mL in the placebo group from week 0 to week 20 (two sample *t*-test* P* = 0.454; mixed model 2-way ANOVA showed a non-significant group effect *P* = 0.7376). Adiponectin increased 0.33 (2.48) μg/mL in the TENS group and 0.25 (4.44) μg/mL in the placebo group (two sample *t*-test* P* = 0.886; mixed model 2-way ANOVA group showed a non-significant group effect *P* = 0.1390). FGF-21 decreased 0.05 (0.19) ng/mL in the TENS group and 0.04 (0.21) ng/mL in the placebo group (two sample *t*-test *P* = 0.752; mixed model 2-way ANOVA showed a significant group effect with *P* = 0.0142).

### Safety outcome

Adverse events are summarized in Table 3 and Supplementary Table S15. About one-third of subjects reported at least one AE during the trial. Number of subjects experiencing at least one AE were 25 (31%) in the TENS group and 28 (35%) in the placebo group. Most reported AEs were mild in severity (TENS: 83%, placebo: 80%). A total of nine serious adverse events (SAEs) were reported during the study, including four events reported from three subjects in the TENS group and five events reported from two subjects in placebo group. None of the SAEs were related to the study device, and no death was reported in this study.

**Table 3:** Adverse events, serious adverse events, and unanticipated adverse device effects.

	TENS (n = 81)	Placebo (n = 79)
Patients with adverse events	25 (31)	28 (35)
Patients with serious adverse events	3 (4)	2 (3)
Patients discontinued due to serious adverse events	0	1 (1)
Total number of adverse events	54	46
Hypoglycemia*	6/54 (11)	14/46 (30)
Severity		
Mild	5/54 (9)	13/46 (28)
Moderate	1/54 (2)	1/46 (2)
Severe	0	0
Hyperglycemia*		
Rescue medication usage	1 (1)^†^	3 (4)^‡^
Unanticipated device adverse effects*		
Redness, swelling, pain	1 (1)	
Hypoglycemia/Pseudo-hypoglycemia		1 (1)

See Supplementary Table S15 for a full listing of adverse events. Data are presented as n (%).

*The number of hypoglycemic events, hyperglycemic events or unanticipated adverse device effects/total adverse events is presented; the percentage of hypoglycemic events, hyperglycemic events or unanticipated adverse device effects/total adverse events is shown in parentheses.

^†^Add-on OAD was the rescue medication in this patient.

^‡^insulin injections were the rescue medications in these patients.

Hypoglycemia, defined as the SMBG levels < 70 mg/dL or when patients suffered from hypoglycemia symptoms, such as weakness, sweating, fast pulse, pallor, dizziness, headache, or loss of consciousness^13^ (see also the Clinical Trial Protocol which can be accessed on the website: https://clinicaltrials.gov/ct2/show/NCT03102424), was the most frequent AE reported, occurring in five (6%) patients in the TENS group vs. nine (11%) patients in the placebo group. Of 100 AEs reported in total, 20 hypoglycemia events were reported by 14 subjects, including 14 events by nine subjects in placebo group (30%) and six events by five subjects in the TENS group (11%). Five subjects had experienced hypoglycemia more than once, including four subjects in the placebo group and one subject in the TENS group. Three (4%) subjects in the placebo group encountered hyperglycemia requiring rescue insulin therapy, and one (1%) subject in the TENS group had hyperglycemia being rescued by add-on OAD. One patient in the TENS group experienced redness, swelling and pain on abdominal wall which completely resolved spontaneously without specific treatment.

Finally, the insulin levels did not differ significantly during the treatment course between the TENS group and the placebo group (Supplementary Table S16). With further analyses of homeostasis model assessment of β-cell function (HOMA-β) and insulin resistance (HOMA-IR) (Supplementary Figure S3), we concluded that islet cell function was not affected by using TENS (two-sample *t*-test showed non-significant difference at week 0 with *P* = 0.410, and at week 20 with *P* = 0.735; mixed model 2-way ANOVA showed a non-significant group effect with *P* = 0.689). Insulin resistance was slightly higher in the TENS group vs. the placebo group before treatment; however, the initially borderline significant difference between the two groups became insignificant at week 20, indicating the potential amelioration of insulin resistance by using TENS (two-sample *t*-test showed a borderline significant pre-treatment difference with *P* = 0.098 and non-significant difference at week 20 with *P* = 0.437; mixed model 2-way ANOVA showed a significant group effect with *P* = 0.002).

## Discussion

This was a phase 3, prospective, double blind, randomized, placebo-controlled, multi-center, pivotal study to investigate the efficacy and safety of TENS in improvement of glycemic control in patients with type 2 diabetes. The primary efficacy endpoint is to compare the HbA_1c_ following a 20-week treatment of TENS vs. placebo. We found that the final mean HbA_1c_ at week 20 is 7.9% in the TENS group and 7.8% in the placebo group, and the mean change of HbA_1c_ from the baseline to week 20 was − 0.2% and − 0.3% in the TENS group and the placebo group, respectively. The secondary endpoints, including the proportions of subject who achieved HbA_1c_ of < 7% at week 20, were similar between the two groups. The mean changes of FPG and 7-point SMBG from baseline to week 20 was greater in the TENS group. However, this relation did not hold on for the primary endpoint, i.e., HbA_1c_ reductions during the study. The disappointing result was possibly related to the already strong effectiveness of current OADs targeting multiple pathogenesis pathways of type 2 diabetes^14,15^, thus leading to the inability of the study to detect the efficacy of TENS in HbA_1c_ reduction on top of the present medications.

However, according to the subgroup analysis, we found that female patients in the TENS group but not the placebo group exhibited a significant HbA_1c_ reduction from baseline. Additionally, HbA_1c_ and FPG from baseline to week 20 significantly decreased in the TENS subgroup of subjects with BMI ≥ 26.9 kg/m^2^. The change was not statistically significant in the corresponding placebo subgroup. Subjects that have had DM duration for more than 9 years in the TENS group had also displayed a significant HbA_1c_ reduction from baseline to week 20. These data suggest TENS might be beneficial for HbA_1c_ and FPG lowering in certain specific clinical conditions.

We further found that the 7-point SMBG maintained a relatively more stable GV in the TENS group compared to the placebo group, as reflected by MAGE reduction, with a greater decline found throughout the study in the TENS group compared to the placebo group. From week 0 to week 20, MAGE decreased 19 mg/dL in the TENS group and 9 mg/dL in the placebo group and became significantly different at week 20 (Fig. 1A and Supplementary Table S10). We further carried out subgroup analysis and found that the trends of greater MAGE decline in the TENS group hold on in different patient subgroups; while female, patients with HbA_1c_ ≥ 8% (the median), and patients with BMI < 26.9 kg/m^2^ (the median) were even more likely to have MAGE reduction than their counterparts.

The significant effect of TENS on MAGE reduction is a post-hoc novel finding of our study. MAGE has long been recognized as an indicator of GV and thus represented as an important part of diabetes control because of the need to reach target HbA_1c_ level while avoiding hypoglycemia^16^. In this study, we measure MAGE by using the 7-point SMBG data to derive the average of each blood glucose increase or decrease (nadir-peak or vice versa)^17^. Several OADs have been shown to decrease GV; most of them belong to DPP4i^18^, SGLT2i^19^, and AGi^20,21^, when compared to SU^18,20^. In our study the use of OADs is well balanced in both groups, except that slightly more patients in the TENS group used SU, which excluded the possibility that differential preference of medication usage results in the beneficial MAGE reduction in the TENS group. Besides, the OADs described above all have respective side effects which might be intolerable or unacceptable by individual patients. On the contrary, our study device exhibited good safety profile and is well tolerated by most patients. Furthermore, GV has been linked to several acute and chronic micro- and macro-vascular complications related to diabetes^10,22–24^. Whether the beneficial effects of TENS on MAGE reduction can be translated to amelioration of short and long-term diabetic complications as well as improvement in quality of life in patients with type 2 diabetes needs to be investigated in the future.

Regarding the safety aspect of the study device, over 80% of TEAEs were mild, and no serious adverse device effects were reported. Hypoglycemia was the most common AE presented with six events occurring in five subjects receiving TENS and 14 events occurring in nine subjects receiving placebo, while hyperglycemia requiring rescue medication was encountered in one subject in the TENS group vs. three subjects in placebo group. Overall, patients in the TENS group experienced less hypoglycemic and hyperglycemic events when compared to placebo, which corroborates with the previous finding that TENS is associated with significant reduction in GV. We propose the underlying mechanism might involve the harmonious regulation of glucagon and insulin secretions associated with TENS, leading to maintenance of optimal glucose homeostasis^25–27^.

Throughout the study, we also found significant decline of CRP from baseline in the TENS group, but not in the placebo group. In addition, CRP and FGF-21 showed significant group effects in the TENS group vs. the placebo group. Although the other biomarkers examined in this study did not show significant change, the decreases in CRP and FGF-21 in the TENS group demonstrated potential improvement in systemic inflammation^28,29^, and fibrosis^30,31^, during the study period. Taken together, we suggest that TENS has the potential benefit to induce reduction of inflammatory cytokine release and end-organ fibrotic damage through avoidance of hypoglycemic and hyperglycemic events, thus might be a safe alternative therapeutic strategy to prevent diabetic complications.

The limitations of this study are listed as below. First, the duration of the study was only 20 weeks, and it is not known whether the MAGE reduction would be sustained or even better for a longer duration as revealed in this study in a time-dependent manner. An extension of the study period to 40 weeks might help to answer the question. Second, since continuous glucose monitoring (CGM)-derived MAGE has been shown to be more accurate than that derived from self-monitoring of blood glucose^20^, further study using CGM to measure MAGE is required to confirm the beneficial effects of TENS on GV. Third, the beneficial results about the exploratory parameters CRP and FGF-21 warrant further study of the underlying mechanisms. Whether this effect is directly from TENS treatment or secondary to the improvement of GV and thus amelioration of oxidative stress needs to be investigated in the future. Finally, this randomized clinical trial was designed to evaluate the potential benefits of TENS on glycemic control; however, we found no statistically significant reduction in major glycemic variables (the primary and secondary endpoints). The significant finding was the difference in MAGE calculated by using SMBG at the end of the trial. Thus, it is important to carry out another randomized clinical trial to confirm the beneficial effects of TENS on GV evaluated by a more elaborate MAGE obtained using CGM before claiming the generalizability including external validity and applicability of the trial findings.

In conclusion, the domestic use of both “Dragon Waves Resonant Home Care” Transcutaneous Electronic Nerve Stimulator (TENS) and placebo (sham TENS ineffective pulse wave) demonstrated a modest effect in glycemic control and were well tolerated without safety concerns in patients with type 2 diabetes. Nonetheless, for the primary endpoint, TENS did not demonstrate a statistically difference in the HbA_1c_ reduction as compared to placebo. The borderline significant reduction of MAGE and the markedly significant difference in MAGE at the end of the study derived from the 7-point SMBG, and of the exploratory biomarkers CRP and FGF-21 in the TENS group as compared to the placebo group warrants further follow-up study to confirm the potential beneficial effects of TENS on GV, inflammation, and fibrosis in patients with type 2 diabetes.

## Methods

### Study design and participants

This was a multi-center, prospective, double blind, randomized, placebo-controlled trial of transcutaneous electrical nerve stimulator (TENS) to improve glycemic control in patients with type 2 diabetes (ClinicalTrials.gov Identifier: NCT03102424). The study and all experimental protocols were approved by Institutional Review Boards of the six joining hospitals (National Taiwan University Hospital, National Cheng Kung University Hospital, Taipei Medical University, Chi Mei Medical Center, Far Eastern Memorial Hospital, and Ditmanson Chia-Yi Christian Hospital), and written informed consent was obtained from each study participant. All experiments were carried out in accordance with relevant guidelines and regulations.

Subjects with type 2 diabetes who met all the inclusion but none of the exclusion criteria were randomized with a 1:1 allocation into either of the 2 groups below: 1. TENS, 2. placebo (sham TENS delivering ineffective pulse wave). The inclusion criteria, exclusion criteria and sample size determination are detailed in the Methods Section in Supplementary Material. A schematic diagram of study design is depicted in Supplementary Fig. S1. The name of the trial registry was “A Prospective, Double Blind, Randomized, Placebo-controlled Trial of Transcutaneous Electrical Nerve Stimulator (DW1330) to Improve Blood Glucose Control in Patients with Type 2 Diabetes”. The full date of first registration is 05/04/2017, and the registration number of the clinical trial is TRWRDM1604001.

### Study intervention

Study visits occurred every 2 or 4 weeks depending on the study phase. The study device “Dragon Waves Resonant Home Care” Transcutaneous Electronic Nerve Stimulator (Taiwan Resonant Waves Research Corporation, Taipei, Taiwan) is a small portable, battery-operated device equipped with wires and 2 patches, and the impulses are sent through wires to patches which are placed on bilateral abdominal wall. The study device used full-frequency wave resonant technology with mixed frequencies ranging from 1 to 20,000 Hz in the TENS group, and from 1 to 30 Hz in the placebo group, respectively. Both the mixed frequency sets were predefined and composed of monophasic square pulse wave with 50% duty cycle. The amplitude of the pulse wave was 7.2 Vpp in average. Patients with surgical or traumatic scars in the abdominal wall were excluded from the study to avoid any interference in the electrical conduction path. The specific location of the stimulation electrode is based on human anatomy to identify the stimulation points (for further details please refer to eAppendix in Supplementary Material).

In pre-clinical setting, DW1330 has been tested in a mouse model of diabetes mellitus. The study is reported in accordance with ARRIVE guidelines (https://arriveguidelines.org). A pilot study on 40 human subjects with type 2 diabetes mellitus has been conducted. Both the animal and human studies are detailed in eAppendix in Supplementary Material.

### Outcome assessments

Primary efficacy variable was the change in HbA_1c_ after 20 weeks of treatment. Secondary efficacy variables included 1. percentage of subjects who achieved HbA_1c_ target of < 7%; 2. change in FPG; 3. changes in mean 7-point SMBG. Safety assessments were as follows, 1. incidence of TEAEs; 2. change in lipid profile and vital signs; 3. percentage of subjects who used rescue medication(s); 4. time to hyperglycemia rescue; 5. frequency and severity of hypoglycemia at each scheduled visit. For post-hoc analysis after the data were seen, as an additional outcome, MAGE was calculated by using the SMBG data to represent the glycemic variability (GV).

We also measured the changes in biomarkers C-reactive protein (CRP), adiponectin, tumor necrosis factorα (TNF-α), fibroblast growth factor-21 (FGF-21), as the exploratory assessments. CRP was determined by latex enhanced immunoturbidimetric assay (ADVIA 1800, ADVIA Chemistry XPT, Dimension EXL, SIEMENS). Adiponectin and TNF-α were measured by enzyme-linked immunosorbent assay (R&D Systems). FGF-21 was quantified by using enzyme-linked immunosorbent assay kits according to manufacturer’s instructions (Duoset human FGF21 ELISA kit, R&D systems, Inc., Minneapolis, MN, USA).

### Measurements and definitions

There were three analysis populations: intention-to-treat (ITT) population, per-protocol (PP) population, and safety population. Both ITT and PP populations were applied to efficacy analyses, while safety population was used in the analysis of safety variables. Definition of the three populations is detailed in the Methods section in Supplementary Material.

### Statistical analysis

The statistical analysis and data management including double data entry, data verification, data validation was performed periodically after receiving case report forms from sites. The statistical analyses were conducted using SAS^®^ software (SAS^®^ Institute Inc., USA, version 9.4) and GraphPad^®^ software (GraphPad Prism^®^ Inc., San Diego, California, USA, version 9.1.0). Two-sample *t*-test was conducted for continuous variable; if normal assumption was violated, Wilcoxon rank sum test would be applied. Chi-square test or Fisher’s exact test were conducted for categorical variable. After the data were disclosed, because of the apparently lower rate of hypoglycemia and hyperglycemia requiring rescue medications experienced by the TENS group when compared to placebo, we also carried out post-hoc analyses on MAGE in addition to the prespecified primary and secondary outcomes. Mixed model two-way analysis of variance (2-way ANOVA) was used to assess the effects of group allocation (TENS vs. placebo), time (before vs. after treatment), and their interaction (group*time) on MAGE and the exploratory assessments. All values are given as means (standard deviation, SD). unless stated otherwise. After the data were disclosed, because of the apparently lower rate of hypoglycemia and hyperglycemia requiring rescue medications experienced by the TENS group when compared to placebo, we also carried out post-hoc analyses on MAGE in addition to the prespecified primary and secondary outcomes.

## Supplementary Information

Supplementary Information.

## Data Availability

The data that support the findings of this study are available from Bestat Pharmaservices Corporation, but restrictions apply to the availability of these data, which were used under license for the current study, and so are not publicly available. Data are however available from the corresponding author upon reasonable request and with permission of Bestat Pharmaservices Corporation.
