# Supplementary material for: A randomized trial on the effect of transcutaneous electrical nerve stimulator on glycemic control in patients with type 2 diabetes
Source: Sci Rep. 2023 Feb 15;13:2662. doi: 10.1038/s41598-023-29791-7 (PMC9932095; doi:10.1038/s41598-023-29791-7)
Supplement: Supplementary file 1 — Supplementary Information. [file 41598_2023_29791_MOESM1_ESM.pdf]

Supplementary Material

Lu JY, Ou HY, Wu, CZ, et al.

A Randomized Trial on the Effect of Transcutaneous Electrical Nerve Stimulator on Glycemic Control in Patients with Type 2 Diabetes

Methods, Tables and Appendix

This supplementary material has been provided by the authors to give readers additional information about their work.

Methods

The full trial protocol can be accessed on the website (https://clinicaltrials.gov/ct2/show/NCT03102424).

Our Previous animal studies have proven the electrical stimulation mode consisting of a specific combination of different frequencies can relieve diabetes- related symptoms without significant side effects and risks of local injuries. With this patented electrical stimulation, we then conducted the clinical case study, the pilot clinical study, and the pivotal randomized double blind clinical study.

At the beginning of our electrical stimulation development, to ensure safety, we set up the upper limit of the electrical current to be 5 mA according to IEEE's safety standards[1]. From our experiments, we found the actual voltage output to be around 7.2V accordingly.

As we are applying our electrical stimulation to treat type 2 diabetes, and type 2 diabetes is a metabolic disease related to pancreas and digestive system, thus, we have selected our stimulation site to be at the abdomen area.

Reference

1. Lloyd B. Gordon, Laura Cartelli, and Nicole Graham. A complete electrical shock hazard classification system and its application. *IEEE Transactions on Industry Applications,* 2018, 54(6), p. 6554 – 6565.

Inclusion criteria

1. Male and female patients aged 30 through 80 years.
2. Type 2 diabetes patients on stable OADs for more than 3 months and can maintain stable during study maintenance period.
3. Subjects with HbA1c between 7.5 and 10% inclusive.
4. Subjects who are able and willing to perform SMBG and self-administration of study device for the entire trial period.
5. Subjects who are able and willing to keep a diary.
6. Subjects who are able and willing to sign informed consent and return for follow-up assessments.

Exclusion criteria

1. Subject has had any of the following new diagnoses within 1 year of screening: myocardial infarction, coronary artery bypass surgery, coronary artery stenting, transient ischemic attack, cerebrovascular accident, angina, congestive heart failure (NYHA III-IV), ventricular rhythm disturbances or thromboembolic disease.
2. Subjects with prior pancreatitis.
3. Subjects with insulin therapy (except for short term uses no longer than 7 days) or

injectables within 3 months.

4. Subject has had any of the following new diagnoses within 1 year of screening: myocardial infarction, coronary artery bypass surgery, coronary artery stenting, transient ischemic attack, cerebrovascular accident, angina, congestive heart failure (NYHA III-IV), ventricular rhythm disturbances or thromboembolic disease.
5. Subjects with prior pancreatitis.
6. Subjects with insulin therapy (except for short term uses no longer than 7 days) or injectables within 3 months.
7. Woman with a positive pregnancy test, planning to become pregnant during screening, active treatment, or the follow up period, breastfeeding, or judged to be using inadequate contraceptive methods.
8. Subjects who underwent previous intra-abdominal, GI tract surgery or a major abdominal trauma within 6 months prior to screening visit.
9. Subjects with other implanted electrical stimulation devices.
10. Subject has any unresolved adverse skin condition in device placement.
11. ALT/AST greater than 3 x upper limit of the institution's normal range (ULN) and/or total bilirubin ≥ 2.0 x ULN, active liver disease (other than nonalcoholic hepatic steatosis), including chronic active hepatitis B or C, hepatic cirrhosis, primary biliary cirrhosis, or active symptomatic gallbladder disease.
12. Subjects with moderate or severe renal impairment (serum creatinine ≥133 μmol/L in male or ≥124 μmol/L in female patients and urine microalbumin-creatinine ratio [ACR) >33.9 mg/mmol), conditions of congenital renal glucosuria, unstable or rapid progressing renal disease.
13. Subjects with blood dyscrasias or any disorders causing hemolysis or unstable red blood cells or any other clinically significant hematological disorder (such as aplastic anemia, myeloproliferative or myelodysplastic syndromes, thrombocytopenia, coagulopathy).
14. Subjects with acute metabolic complications (such as ketoacidosis, lactic acidosis or hyperosmolar), proliferative diabetic retinopathy or macular edema within 6 months before screening.
15. Subjects with a history of malignancy ≤5 years prior to screening, except for adequately treated basal or squamous cell skin cancer or in situ cervical cancer.
16. Subjects with a history of alcohol or drug abuse within 1 year prior to screening.
17. Subjects who received another investigational agent within 30 days prior to screening.
18. Subjects who are unlikely to be available for follow-up as specified in the protocol.
19. Subjects with a past or present psychiatric condition that may impair his or her ability to comply with the study procedures.
20. Subjects with conditions that, in the judgment of the investigator, precludes successful participation to the study.

Sample size determination

The primary efficacy endpoint is to compare the change of HbA_{1c} following a 20-week treatment of DW1330 versus placebo. According to prior published clinical reference and unpublished data on DW1330, we assumed that the mean HbA_{1c} reduction is 5.5 mmol/mol. The evaluable subject number should be at least 64 subjects for each group under these conditions: the one-sided significance level is 0.025, the power is 80%, the difference between control and experimental group is 5.5 mmol/mol, and the standard deviation of both treatment groups is 10.9 mmol/mol. Considering a 20% drop-out rate, a sample size of 80 subjects should be enrolled in each group, and a total of 160 subjects will be recruited into this study.

Study visits

At randomization visit study device was dispensed at the study site of six hospitals: National Taiwan University Hospital, National Cheng Kung University Hospital, Shuang Ho Hospital, Chi Mei Medical Center, Far Eastern Memorial Hospital, and Ditmanson Chia-Yi Christian Hospital. During the treatment period visits, all treatment emergent adverse events (TEAEs) as well as vital signs, changes to concomitant medications, efficacy evaluations were recorded, and blood and urine samples were collected. Investigators, site staff, subjects, and the study team were blinded to the device assigned. The end-of-treatment visit and the last estimation of glycemia occurred at week 20 (visit 8) for all subjects. In addition, a 2-week follow-up occurred to collect safety data after the device was returned. The final visit was at week 22 (visit 9). Subjects were encouraged to complete all planned visits regardless of their adherence to study device administration. A permuted block randomization method with 1: 1 ratio was employed to randomize subjects into one of the two arms as follows, (1) DW1330 treatment group or (2) Placebo group. The study duration was three years; and dates defining the periods of recruitment and follow-up were 19 July 2017 (the recruitment of the first case) and 15 July 2020 (the follow-up of the last case).

Randomization

A randomization code list and the random allocation sequence were generated by Bestat Pharmaservices Corp. and provided to Taiwan Resonant Waves Research Corp. to pack and label the study devices. Randomization data were not accessible by anyone else involved in the study. The corresponding study groups of individual subjects were recorded in a sealed envelope and kept strictly confidential until the time of data lock.

Study machine

The study device, "Dragon Waves Resonant Home Care" Electronic Nerve Stimulator (DW1330), indicated to relieve pain, reduce the sensitivity of peripheral nerve system, and stimulate blood circulation, has been approved for marketing in Taiwan. DW1330 uses full-frequency wave resonant technology while the principle is like other electrical stimulation devices, such as transcutaneous electrical nerve stimulator (TENS), low frequency therapeutic device, or middle frequency stimulator. Subjects received their study device and applicable training and were instructed to use the device 1 hour after dinner by attaching the patches to the left and right side, around 5-10 cm from the navel as shown in eAppendix. The frequency of device use was one hour per day, 5 days/week.

Definition of study populations

These analysis populations were defined as following: ITT population is the randomized subjects who met all the inclusion and none of the exclusion criteria, used at least one time of the study device and had at least one post-randomization measurement. PP population is the subset of the ITT population who completed 20 weeks of treatment without major protocol violation. The definitions of major violations include: 1. device compliance less than 70%; 2. violation of eligibility criteria; 3. use of prohibited medications. Safety population is all randomized subjects who used at least one time of the study device.

Data management

Data management and statistical analysis was conducted by Bestat Pharmaservices Corp. The data manager generated queries using data clarification forms to the site personnel when there were any potential errors, omissions, or unlikely values in the data. After confirming all the discrepancies and resolving all queries, the database was locked by the data manager in line with the standard operating procedures.

Table S1. Summary of protocol deviations by subjects

Description	Total	TENS	Placebo
Total number of protocol deviations	85	45	40
IP not compliance	42	22	20
Out of allow window	6	3	3
OADs dosage change during maintenance period (V5-V8)	4	2	2
Did not follow the study procedure	2	2	0
Forgot to record SMBG	15	7	8
Lost IP supply	2	2	0
Blood sample clotting	1	0	1
Not fasting for blood test	1	1	0
Not enough sample for FGF-21 analysis	1	1	0
Recorded on the wrong version diary card	1	0	1
Recorded on the wrong version CRF	7	4	3
12-lead ECG out of allow window	1	0	1
Non-systemic steroid long-term use	1	1	0
Could not complete the study	1	0	1

Abbreviations: IP, investigational product; OADs, oral antidiabetic drugs; V, visit; FGF-21, fibroblast growth factor 21; CRF, case report form; ECG, electrocardiography.

Table S2. Demographic characteristics for the PP population

	TENS	Placebo	*P* value
No.	74	73	
Age, years	60 (10)	58 (10)	0.406
Gender, male, n (%)	42 (57)	45 (62)	0.547
Height, cm	163 (10)	164 (9)	0.679
Weight, kg	72 (16)	74 (15)	0.534
BMI, kg/m^2	26.8 (4.5)	27.3 (4.3)	0.545
HbA1c, %	8.0 (0.5)	8.1 (0.5)	0.914
FPG, mg/dL	168 (51)	156 (39)	0.129
Mean 7-point SMBG, mg/dL	183 (33)	180 (29)	0.568
MAGE, mg/dL	84 (33)	88 (30)	0.255
SBP, mmHg	128 (16)	127 (14)	0.730
DBP, mmHg	78 (10)	78 (9)	0.545
TC, mg/dL	160 (31)	163 (31)	0.584
TG, mg/dL	159 (96)	156 (97)	0.850
LDL-C, mg/dL	89 (24)	94 (23)	0.232
HDL-C, mg/dL	46 (13)	46 (12)	0.828
OADs prescribed			
Metformin, n (%)	70 (95)	72 (99)	0.366*
SU, n (%)	9 (12)	2 (3)	0.056*
DPP4i, n (%)	24 (32)	22 (30)	0.903
SGLT2i, n (%)	15 (20)	23 (32)	0.171
TZD, n (%)	17 (23)	13 (18)	0.567
AGi, n (%)	6 (8)	4 (6)	0.745*

Abbreviations: BMI, body mass index; HbA1c, glycosylated hemoglobin; FPG, fasting plasma glucose; SMBG, self-monitoring blood glucose; MAGE, mean amplitude of glycemic excursion; SBP, systolic blood pressure; DBP, diastolic blood pressure; TC, total cholesterol; TG, triglyceride; LDL-C, low-density lipoprotein cholesterol; HDL-C, high-density lipoprotein cholesterol; OADs, oral anti-hyperglycemic drugs; SU, sulfonylurea; DPP4i, dipeptidyl peptidase 4 inhibitor; SGLT2i, sodium-glucose cotransporter 2 inhibitor; TZD, thiazolidinedione; AGi, α-glucosidase inhibitor. *Fisher's exact test was conducted for categorical variable. Data are presented as means (SD). Two-sample *t*-test was conducted for continuous variable; Chi-square test or Fisher's exact test were conducted for categorical variable.

Table S3. Study treatment compliance in ITT and PP populations

Population	TENS	Placebo
ITT population		
N	78	77
Mean	109	107
SD	18	24
Median	103	104
Range (min, max)	21, 140	0, 147
95% CI	105, 114	101, 113
P value	0.481	
PP population		
N	74	73
Mean	111	111
SD	15	15
Median	103	104
Range (min, max)	73, 140	90, 147
95% CI	107, 114	108, 115
P value	0.859	

Two-sample *t*-test was conducted. SD, standard deviation. Compliance rate (%): Total usage time of the investigational product (IP) during the trial (min)/6000 (min)*100.

Table S4. Mean change in glycemic parameters

Summary of mean change in glycemic parameters from week 0 to week 20 in ITT population

	Time (week)	TENS (n=78)	Placebo (n=77)	*P* value
HbA_{1c}, %	2	-0.09 (0.19)<br>[-0.13, -0.05]	-0.09 (0.29)<br>[-0.15, -0.02]	0.503
	4	-0.19 (0.28)<br>[-0.25, -0.12]	-0.18 (0.39)<br>[-0.27, -0.09]	0.700
	8	-0.30 (0.41)<br>[-0.40, -0.21]	-0.30 (0.49)<br>[-0.38, -0.15]	0.606
	12	-0.32 (0.61)<br>[-0.46, -0.18]	-0.30 (0.66)<br>[-0.45, -0.15]	0.859
	16	-0.29 (0.62)<br>[-0.43, -0.15]	-0.34 (0.74)<br>[-0.50, -0.17]	0.863
	20	-0.24 (0.64)<br>[-0.39, -0.09]	-0.33 (0.82)<br>[-0.52, -0.15]	0.821
FPG, mg/dL	2	-11 (38)<br>[-20, -2]	-6 (34)<br>[-14, 2]	0.937
	4	-13 (34)<br>[-21, -6]	-4 (38)<br>[-12, 5]	0.285
	8	-14 (46)<br>[-24, -3]	-12 (46)<br>[-22, -2]	0.575
	12	-11 (41)<br>[-20, -1]	-6 (51)<br>[-17, 6]	0.522
	16	-11 (44)<br>[-20, -1]	-11 (49)<br>[-22, 0]	0.997
	20	-11 (45)<br>[-22, -1]	-4 (56)<br>[-16, 9]	0.369
Mean 7-point SMBG, mg/dL	4	-8 (29)<br>[-15, -1]	-5 (34)<br>[-12, 3]	0.497
	12	-8 (37)<br>[-17, 0]	-8 (36)<br>[-16, 1]	0.518
	20	-11 (34)<br>[-19, -3]	-8 (36)<br>[-17, 0]	0.730
MAGE, mg/dL	4	-3 (40)<br>[-13, 6]	-5 (44)<br>[-15, 5]	0.641
	12	-10 (12)<br>[-19, -1]	-9 (7)<br>[-20, 1]	0.866

	20	-19 (39)<br>[-28, -10]	-9 (43)<br>[-19, 2]	0.087

Abbreviations: HbA_{1c}, glycosylated hemoglobin; FPG, fasting plasma glucose; SMBG, self-monitoring blood glucose; MAGE, mean amplitude of glycemic excursion. Data are presented in means (SD) and [95% CI]. Two-sample *t*-test was conducted.

Summary of mean change in glycemic parameters from week 0 to week 20 in PP population

	Time (week)	TENS (n=74)	Placebo (n=73)	*P* value
HbA1c, %	2	-0.09 (0.19)<br>[-0.14, -0.05]	-0.09 (0.30)<br>[-0.16, -0.02]	0.552
	4	-0.19 (0.28)<br>[-0.26, -0.13]	-0.17 (0.39)<br>[-0.26, -0.08]	0.789
	8	-0.32 (0.41)<br>[-0.42, -0.22]	-0.27 (0.50)<br>[-0.38, -0.15]	0.480
	12	-0.34 (0.61)<br>[-0.48, -0.20]	-0.31 (0.68)<br>[-0.46, -0.15]	0.768
	16	-0.31 (0.63)<br>[-0.46, -0.16]	-0.34 (0.75)<br>[-0.52, -0.17]	0.715
	20	-0.27 (0.66)<br>[-0.43, -0.12]	-0.34 (0.84)<br>[-0.54, -0.14]	0.926
FPG, mg/dL	2	-11 (39)<br>[-20, -2]	-6 (34)<br>[-14, 2]	0.995
	4	-14 (35)<br>[-22, -6]	-4 (38)<br>[-13, 5]	0.292
	8	-14 (47)<br>[-25, -3]	-13 (47)<br>[-24, -2]	0.640
	12	-11 (42)<br>[-20, -1]	-6 (52)<br>[-18, 6]	0.578
	16	-11 (45)<br>[-22, -1]	-12 (50)<br>[-24, -1]	0.956
	20	-12 (46)<br>[-23, -2]	-4 (57)<br>[-18, 9]	0.396
Mean 7-point SMBG, mg/dL	4	-8 (30)<br>[-15, -1]	-5 (34)<br>[-13, 2]	0.577
	12	-8 (37)<br>[-17, 1]	-8 (37)<br>[-16, 1]	0.550
	20	-11 (34)	-8 (37)	0.671

		[-19, -3]	[-17, 0]	
MAGE, mg/dL	4	-3 (41)	-6 (43)	0.641
		[-13, 6]	[-16, 5]	
	12	-8 (39)	-9 (45)	0.857
		[-17, 1]	[-20, 1]	
	20	-18 (39)	-9 (43)	0.140
		[-27, -9]	[-19, 2]	

Abbreviations: HbA1c, glycosylated hemoglobin; FPG, fasting plasma glucose; SMBG, self-monitoring blood glucose; MAGE, mean amplitude of glycemic excursion. Data are presented as means (SD) and [95% CI]. Two-sample *t*-test was conducted.

Table S5. HbA$_{1c}$ at each visit for the ITT and PP populations

Summary of HbA1c by visit (ITT population)

HbA$_{1c}$, %	TENS		Placebo
Screening			
N	78		77
Mean	8.0		8.1
SD	0.5		0.5
95% CI	8.0, 8.2		8.0, 8.2
P value*		0.789	
Visit 2 (Week 0)			
N	77		77
Mean	8.1		8.1
SD	0.6		0.6
95% CI	8.0, 8.2		7.9, 8.2
P value*		0.989	
Visit 3 (Week 2)			
N	78		75
Mean	8.0		8.0
SD	0.6		0.7
95% CI	7.9, 8.1		7.8, 8.2
P value*		0.998	
Visit 4 (Week 4)			
N	78		75
Mean	8.0		8.0
SD	0.6		0.8
95% CI	7.8, 8.0		7.7, 8.1
P value*		0.796	
Visit 5 (Week 8)			
N	77		74
Mean	7.8		7.8
SD	0.6		0.7
95% CI	7.7, 7.9		7.6, 8.0
P value*		0.983	
Visit 6 (Week 12)			
N	76		74
Mean	7.8		7.8
SD	0.7		0.8
95% CI	7.6, 7.9		7.6, 8.0

HbA_{1c}, %	TENS		Placebo
P value*		0.886	
Visit 7 (Week 16)			
N	77		74
Mean	7.8		7.7
SD	0.7		0.8
95% CI	7.6, 8.0		7.6, 7.9
P value*		0.820	
Visit 8 (Week 20)			
N	77		73
Mean	7.8		7.8
SD	0.8		0.8
95% CI	7.7, 8.0		7. 6, 7.9
P value*		0.540	

Two-sample *t*-test was conducted for continuous variable. *If normal assumption were violated, Wilcoxon rank sum test would be applied. SD, standard deviation; CI, confidence interval.

Summary of HbA1c by visit (PP population)

HbA_{1c}, %	TENS		Placebo
Screening			
N	74		73
Mean	8.0		8.1
SD	0.5		0.5
95% CI	8.0, 8.2		8.0, 8.2
P value*		0.914	
Visit 2 (Week 0)			
N	73		73
Mean	8.1		8.1
SD	0.6		0.6
95% CI	8.0, 8.3		8.0, 8.2
P value*		0.989	
Visit 3 (Week 2)			
N	74		73
Mean	8.0		8.0
SD	0.6		0.7
95% CI	7.9, 8.2		7.8, 8.2
P value*		0.967	

HbA_{1c}, %	TENS		Placebo
Visit 4 (Week 4)			
N	74		73
Mean	7.9		7.9
SD	0.6		0.8
95% CI	7.8, 8.1		7.7, 8.1
P value*		0.924	
Visit 5 (Week 8)			
N	74		73
Mean	7.8		7.8
SD	0.6		0.7
95% CI	7.7, 7.9		7.7, 8.0
P value*		0.867	
Visit 6 (Week 12)			
N	73		73
Mean	7.8		7.8
SD	0.7		0.8
95% CI	7.6, 7.9		7.6, 7.8
P value*		0.812	
Visit 7 (Week 16)			
N	74		73
Mean	7.8		7.8
SD	0.7		0.8
95% CI	7.6, 8.0		7.6, 7.9
P value*		0.942	
Visit 8 (Week 20)			
N	74		73
Mean	7.8		7.8
SD	0.8		0.8
95% CI	7.6, 8.0		7. 6, 7.9
P value*		0.679	

Two-sample *t*-test was conducted for continuous variable. *If normal assumption were violated, Wilcoxon rank sum test would be applied. SD, standard deviation; CI, confidence interval.

Table S6. Subjects achieving HbA1c <7.0% by visit.

Summary of proportion of subjects achieving HbA1c <7.0% by visit (ITT population)

	TENS N=78		Placebo N=77
Screening			
HbA1c <7%	78 (100)		77 (100)
P value		NA	
Visit 2 (Week 0)			
HbA1c ≥7%	77 (100)		77 (100)
P value		NA	
Visit 3 (Week 2)			
HbA1c ≥7%	78 (100)		75 (100)
P value		NA	
Visit 4 (Week 4)			
HbA1c ≥7%	78 (100)		74 (99)
HbA1c <7%	0 (0)		1 (1)
P value		0.306	
Visit 5 (Week 8)			
HbA1c ≥7%	74 (96)		67 (90)
HbA1c <7%	3 (4)		7 (10)
P value		0.169	
Visit 6 (Week 12)			
HbA1c ≥7%	71 (93)		62 (84)
HbA1c <7%	5 (7)		12 (16)
P value		0.063	
Visit 7 (Week 16)			
HbA1c ≥7%	72 (93)		65 (88)
HbA1c <7%	5 (7)		9 (12)
P value		0.230	
Visit 8 (Week 20)			
HbA1c ≥7%	69 (90)		65 (89)
HbA1c <7%	8 (10)		8 (11)
P value		0.910	

Chi-square test was conducted. Data are presented as n (%).

Summary of proportion of subjects achieving HbA1c <7.0% by visit (PP population)

	TENS N=78		Placebo N=77
Statistics	TENS N=74		Placebo N=73
Screening			
HbA$_{1c}$ ≥7%	74 (100)		73 (100)
P value		NA	
Visit 2 (Week 0)			
HbA1c ≥7%	73 (100)		73 (100)
P value		NA	
Visit 3 (Week 2)			
HbA$_{1c}$ ≥7%	74 (100)		73 (100)
P value		NA	
Visit 4 (Week 4)			
HbA$_{1c}$ ≥7%	74 (100)		72 (99)
HbA$_{1c}$ <7%	0 (0)		1 (1)
P value		0.312	
Visit 5 (Week 8)			
HbA$_{1c}$ ≥7%	71 (96)		66 (90)
HbA$_{1c}$ <7%	3 (4)		7 (10)
P value		0.183	
Visit 6 (Week 12)			
HbA$_{1c}$ ≥7%	68 (93)		61 (84)
HbA$_{1c}$ <7%	5 (7)		12 (16)
P value		0.071	
Visit 7 (Week 16)			
HbA$_{1c}$ ≥7%	69 (93)		64 (88)
HbA$_{1c}$ <7%	5 (7)		9 (12)
P value		0.250	
Visit 8 (Week 20)			
HbA$_{1c}$ ≥7%	66 (89)		65 (89)
HbA$_{1c}$ <7%	8 (11)		8 (11)
P value		0.977	

Chi-square test was conducted. Data are presented as n (%).

Table S7. FPG at each visit for the ITT and PP populations

Summary of FPG by visit (ITT population)

FPG, mg/dL	TENS<br>N = 78		Placebo<br>N = 77
Visit 2 (Week 0)			
N	78		77
Mean	168		155
SD	50		39
95% CI	157, 180		146, 164
P value*		0.086	
Visit 3 (Week 2)			
N	78		75
Mean	157		149
SD	41		40
95% CI	148, 167		140, 158
P value*		0.072	
Visit 4 (Week 4)			
N	78		75
Mean	155		152
SD	42		43
95% CI	146, 165		142, 162
P value*		0.397	
Visit 5 (Week 8)			
N	77		74
Mean	155		143
SD	43		34
95% CI	145, 164		135, 151
P value*		0.121	
Visit 6 (Week 12)			
N	76		74
Mean	158		149
SD	38		37
95% CI	149, 167		141, 158
P value*		0.095	
Visit 7 (Week 16)			
N	77		74
Mean	158		144
SD	44		35

FPG, mg/dL	TENS<br>N = 78		Placebo<br>N = 77
95% CI	148, 168		136, 152
P value*		0.122	
Visit 8 (Week 20)			
N	77		73
Mean	157		151
SD	45		42
95% CI	147, 167		141, 161
P value*		0.351	

Abbreviations: SD, standard deviation; CI, confidence interval. Two-sample *t*-test was conducted for continuous variable. *If normal assumption was violated, Wilcoxon rank sum test would be applied.

Summary of FPG by visit (PP population)

FPG (mmol/L)	TENS<br>N = 74		Placebo<br>N = 73
Visit 2 (Week 0)			
N	74		73
Mean	168		156
SD	51		39
95% CI	157, 180		146, 165
P value*		0.129	
Visit 3 (Week 2)			
N	74		73
Mean	157		149
SD	42		40
95% CI	147, 167		140, 159
P value*		0.100	
Visit 4 (Week 4)			
N	74		73
Mean	154		151
SD	43		44
95% CI	144, 164		141, 161
P value*		0.420	
Visit 5 (Week 8)			
N	74		73
Mean	154		142

FPG (mmol/L)	TENS<br>N = 74		Placebo<br>N = 73
SD	44		35
95% CI	144, 164		134, 150
P value*		0.154	
Visit 6 (Week 12)			
N	73		73
Mean	158		149
SD	39		37
95% CI	149, 1671		141, 158
P value*		0.115	
Visit 7 (Week 16)			
N	74		73
Mean	157		143
SD	45		35
95% CI	147, 167		135, 151
P value*		0.159	
Visit 8 (Week 20)			
N	74		73
Mean	156		151
SD	46		42
95% CI	145, 167		141, 161
P value*		0.492	

Abbreviations: SD, standard deviation; CI, confidence interval. Two-sample *t*-test was conducted for continuous variable. *If normal assumption was violated, Wilcoxon rank sum test would be applied.

Table S8. Summary of significant differences in subgroup analysis- the within group mean change of glycemic parameters in ITT or PP population

Differences within the group of mean change of HbA1c (%) from baseline (ITT)-Female

Statistics	TENS<br>N = 35	Placebo<br>N = 30
Mean change	-0.19	-0.14
SD	0.49	0.61
95% CI	-0.35, -0.02	-0.37, 0.08
P value	0.0320*	0.2050

Abbreviations: SD, standard deviation; CI, confidence interval. Paired *t*-test was conducted. The last observation carried forward (LOCF) method would be adopted to establish this missing efficacy value. *Statistically significant.

Differences within the group of mean change of HbA1c (%) from baseline (ITT)-Male

Statistics	TENS<br>N = 42	Placebo<br>N = 47
Mean change	-0.28	-0.45
SD	0.78	0.91
95% CI	-0.53, -0.04	-0.72, -0.18
P value	0.0235*	0.0015*

Abbreviations: SD, standard deviation; CI, confidence interval. Paired *t*-test was conducted. The last observation carried forward (LOCF) method would be adopted to establish this missing efficacy value. *Statistically significant.

Differences within the group of mean change of HbA1c (%) from baseline (PP)-Female

Statistics	TENS<br>N = 32	Placebo<br>N = 28
Mean change	-0.26	-0.15
SD	0.47	0.63
95% CI	-0.43, -0.08	-0.40, 0.09
P value	0.0049*	0.2054

Abbreviations: SD, standard deviation; CI, confidence interval. Paired *t*-test was conducted. The last observation carried forward (LOCF) method would be adopted to establish this missing efficacy value. *Statistically significant.

Differences within the group of mean change of HbA1c (%) from baseline (PP)-Male

Statistics	TENS<br>N = 42	Placebo<br>N = 45

Mean change	-0.28	-0.46
SD	0.78	0.93
95% CI	-0.53, -0.04	-0.74, -0.18
P value	0.0235*	0.0021*

Abbreviations: SD, standard deviation; CI, confidence interval. Paired *t*-test was conducted. The last observation carried forward (LOCF) method would be adopted to establish this missing efficacy value. *Statistically significant.

Differences within the group of mean change of FPG (mg/dL) from baseline (PP)-Female

Statistics	TENS<br>N = 32	Placebo<br>N = 28
Mean change	-17	9
SD	47	52
95% CI	-342, 0	-11, 29
P value	0.0470*	0.3527

Abbreviations: SD, standard deviation; CI, confidence interval. Paired *t*-test was conducted. The last observation carried forward (LOCF) method would be adopted to establish this missing efficacy value. *Statistically significant.

Differences within the group of mean change of HbA1c (%) from baseline (ITT)-BMI ≥26.9 (median)

Statistics	TENS<br>N = 39	Placebo<br>N = 39
Mean change	-0.24	-0.21
SD	0.66	0.89
95% CI	-0.45, -0.02	-0.49, 0.08
P value	0.0309*	0.1513

Abbreviations: SD, standard deviation; CI, confidence interval. Paired *t*-test was conducted. The last observation carried forward (LOCF) method would be adopted to establish this missing efficacy value. *Statistically significant.

Differences within the group of mean change of HbA1c (%) from baseline (PP)-BMI ≥26.9 kg/m^2 (median)

Statistics	TENS<br>N = 37	Placebo<br>N = 37
Mean change	-0.26	-0.20
SD	0.67	0.91
95% CI	-0.49, -0.04	-0.51, 0.10

P value	0.0218*	0.1824

Abbreviations: SD, standard deviation; CI, confidence interval. Paired *t*-test was conducted. The last observation carried forward (LOCF) method would be adopted to establish this missing efficacy value. *Statistically significant.

Differences within the group of mean change of FPG (mg/dL) from baseline (ITT)-BMI ≥26.9 kg/m^2 (median)

Statistics	TENS<br>N = 39	Placebo<br>N = 39
Mean change	-14	-8
SD	40	59
95% CI	-27, 0	-27, 11
P value	0.0425*	0.4009

Abbreviations: SD, standard deviation; CI, confidence interval. Paired *t*-test was conducted. The last observation carried forward (LOCF) method would be adopted to establish this missing efficacy value. *Statistically significant.

Differences within the group of mean change of FPG (mg/dL) from baseline (PP)-BMI ≥26.9 kg/m^2 (median)

Statistics	TENS<br>N = 37	Placebo<br>N = 37
Mean change	-15	-8
SD	41	61
95% CI	-29, -1	-29, 12
P value	0.0325*	0.4043

Abbreviations: SD, standard deviation; CI, confidence interval. Paired *t*-test was conducted. The last observation carried forward (LOCF) method would be adopted to establish this missing efficacy value. *Statistically significant.

Differences within the group of mean change of HbA1c (%) from baseline (ITT)-DM duration >9 years (median)

Statistics	TENS<br>N = 35	Placebo<br>N = 35
Mean change	-0.28	-0.28
SD	0.73	0.92
95% CI	-0.53, -0.03	-0.59, 0.04
P value	0.0282*	0.0837

Abbreviations: SD, standard deviation; CI, confidence interval. Paired *t*-test was conducted.

The last observation carried forward (LOCF) method would be adopted to establish this missing efficacy value. *Statistically significant.

Differences within the group of mean change of HbA1c (%) from baseline (PP)-DM duration >9 years (median)

Statistics	TENS<br>N = 35	Placebo<br>N = 31
Mean change	-0.28	-0.29
SD	0.73	0.97
95% CI	-0.53, -0.03	-0.65, 0.07
P value value	0.0282*	0.1076

Abbreviations: SD, standard deviation; CI, confidence interval. Paired *t*-test was conducted. The last observation carried forward (LOCF) method would be adopted to establish this missing efficacy value. *Statistically significant.

Table S9. Summary of the mean 7-point self-monitoring blood glucose (SMBG) level at each visit for the ITT and PP populations

The mean 7-point SMBG level at each visit for the ITT population

Mean 7-point SMBG, mg/dL	TENS N = 78		Placebo N = 77
Visit 2 (Week 0)			
N	78		76
Mean	184		180
SD	33		28
95% CI	176, 191		174, 187
P value*		0.746	
Visit 4 (Week 4)			
N	77		77
Mean	176		176
SD	32		35
95% CI	168, 183		168, 184
P value*		0.901	
Visit 6 (Week 12)			
N	77		74
Mean	176		173
SD	34		31
95% CI	168, 183		165, 180
P value*		0.697	
Visit 8 (Week 20)			
N	77		73
Mean	173		172
SD	34		34
95% CI	165, 181		164, 180
P value*		0.947	

Abbreviations: SD, standard deviation; CI, confidence interval. Two-sample *t*-test was conducted for continuous variable. *If normal assumption was violated, Wilcoxon rank sum test would be applied.

Mean 7-point SMBG (mg/dL)	TENS N = 74	Placebo N = 73
Visit 2 (Week 0)		
N	74	73
Mean	183	180

Mean 7-point SMBG, mg/dL	TENS<br>N = 78		Placebo<br>N = 77
SD	33		29
95% CI	176, 191		174, 187
P value*		0.568	
Visit 4 (Week 4)			
N	74		73
Mean	175		175
SD	32		34
95% CI	168, 182		167, 183
P value*		0.967	
Visit 6 (Week 12)			
N	74		73
Mean	175		173
SD	35		32
95% CI	167, 183		163, 180
P value*		0.741	
Visit 8 (Week 20)			
N	74		73
Mean	172		172
SD	35		34
95% CI	164, 180		164, 180
P value*		0.910	

Abbreviations: SD, standard deviation; CI, confidence interval. Two-sample *t*-test was conducted for continuous variable. *If normal assumption was violated, Wilcoxon rank sum test would be applied.

Table S10. Summary of MAGE (mean amplitude of glycemic excursion) by visit

MAGE at each visit for the ITT population

MAGE, mg/dL	TENS		Placebo
Visit 2, week 0			
N	78		76
Mean	85		88
SD	33		30
Median	80		85
Range (min, max)	28, 167		21, 178
95% CI	77, 92		81, 95
P value		0.354	
Visit 4, week 4			
N	77		74
Mean	81		84
SD	34		40
Median	78		77
Range (min, max)	15, 174		17, 226
95% CI	74, 89		74, 93
P value		0.710	
Visit 6, week 16			
N	77		73
Mean	75		79
SD	37		33
Median	73		76
Range (min, max)	11, 229		18, 175
95% CI	67, 83		71, 86
P value*		0.376	
Visit 8, week 20			
N	77		73
Mean	66		79
SD	33		33
Median	60		73
Range (min, max)	6, 155		25, 168
95% CI	58, 73		72, 87
P value		0.009*	

Abbreviations: SD, standard deviation; CI, confidence interval. Two sample *t*-test was conducted. If normal assumption was violated, Wilcoxon rank sum test would be applied.
*Statistically significant.

MAGE at each visit for the PP population

MAGE, mg/dL	TENS		Placebo
Visit 2, week 0			
N	74		73
Mean	84		88
SD	33		30
Median	78		84
Range (min, max)	28, 167		21, 178
95% CI	76, 91		81, 95
P value		0.255	
Visit 4, week 4			
N	74		72
Mean	80		83
SD	34		39
Median	77		77
Range (min, max)	15, 174		17, 226
95% CI	73, 88		74, 92
P value		0.692	
Visit 6, week 16			
N	74		72
Mean	75		78
SD	37		34
Median	73		76
Range (min, max)	11, 229		18, 175
95% CI	67, 84		70, 86
P value		0.443	
Visit 8, week 20			
N	74		73
Mean	66		79
SD	34		33
Median	60		73
Range (min, max)	6, 155		25, 168
95% CI	58, 74		72, 87
P value		0.012*	

Abbreviations: SD, standard deviation; CI, confidence interval. Two sample t-test was conducted. If normal assumption was violated, Wilcoxon rank sum test would be applied. *Statistically significant.

Table S11. The events of OAD regimen change in the maintenance period of the study

Group	OAD	Dose	Route	Note
Placebo	Glimepiride<br>Glimepiride	2 mg<br>4 mg	Oral<br>Oral	Dose increased during maintenance period
Placebo	Gliclazide<br>Gliclazide	60 mg<br>30 mg	Oral<br>Oral	Dose reduced during maintenance period due to hypoglycemia event
TENS	Dapagliflozin<br>Dapagliflozin	5 mg<br>10 mg	Oral<br>Oral	Dose increased during maintenance period
TENS	Glimepiride	4 mg	Oral	Added on new OAD during the maintenance period

Abbreviation: OAD: oral antidiabetic drug.

Table S12. Summary of vital signs (Safety population)

	TENS	Placebo
Vital signs at Week 0		
SBP (mmHg)		
N	81	79
Mean	128	127
SD	15	13
95% CI	124, 131	124, 130
P value		0.746
DBP (mmHg)		
N	81	79
Mean	77	78
SD	10	9
95% CI	75, 80	76, 80
P value		0.611
Pulse (beats/min)		
N	81	79
Mean	79	79
SD	11	11
95% CI	77, 81	77, 81
P value		0.982
Respiratory rate (times/min)		
N	81	79
Mean	16	16
SD	2	2
95% CI	16, 17	16, 17
P value		0.870
Body temperature (°C)		
N	81	79
Mean	36.5	36.4
SD	0.3	0.4
95% CI	36.4, 36.6	36.3, 36.5
P value		0.056
Vital signs at Week 20		
SBP (mmHg)		
N	79	75
Mean	127	124
SD	15	13

	TENS		Placebo
95% CI	123, 130		121, 127
P value		0.336	
DBP (mmHg)			
N	79		75
Mean	76		77
SD	10		0
95% CI	74, 78		75, 79
P value		0.572	
Pulse (beats/min)			
N	79		75
Mean	79		79
SD	11		10
95% CI	77, 82		77, 81
P value		0.891	
Respiratory rate (times/min)			
N	79		75
Mean	16		16
SD	2		2
95% CI	16, 17		16, 17
P value		0.992	
Body temperature (°C)			
N	79		75
Mean	36.5		36.4
SD	0.3		0.4
95% CI	36.4, 36.6		36.3, 36.5
P value		0.541	

Abbreviations: DBP, diastolic blood pressure; SBP, systolic blood pressure; SD, standard deviation; CI, confidence interval. Two-sample *t*-test was conducted for continuous variable.

Table S13. The comparisons of lipid profile between groups

Summary of lipid profile change from baseline in ITT population

	TENS (n=78)	Placebo (n=77)
TC		
Mean change	-1	-7
Median	-1	-5
SD	24	26
Range	-91, 57	-113, 45
95%CI	-6, 5	-13, -1
P value	0.814	0.023*
TG		
Mean change	-11	-20
Median	-10	-7
SD	63	84
Range	-204, 249	-431, 171
95%CI	-26, 3	-39, 0
P value	0.116	0.049*
LDL-C		
Mean change	-1	-5
Median	0	-1
SD	20	22
Range	-83, 47	-75, 47
95%CI	-5, 4	-10, 0
P value	0.698	0.068
HDL-C		
Mean change	2	1
Median	1	1
SD	7	7
Range	(-12, 22	-19, 20
95%CI	0, 3	-1, 2
P value	0.016*	0.320

Abbreviations: TC, total cholesterol; TG, triglyceride; LDL-C, low density lipoprotein cholesterol; HDL-C, high density lipoprotein cholesterol; SD, standard deviation; CI, confidence interval. **P* <0.05. Paired *t*-test was conducted.

Summary of lipid profile change from baseline in PP population

	TENS (n=74)	Placebo (n=73)
TC		

Mean change	-1	-7
Median	-1	-5
SD	24	26
Range	-91, 57	-113, 45
95%CI	-6, 5	-13, -1
P value	0.754	0.023*
TG		
Mean change	-11	-20
Median	-10	-7
SD	64	84
Range	-204, 249	-431, 171
95%CI	-26, 4	-39, 0
P value	0.137	0.049*
LDL-C		
Mean change	-1	-5
Median	0	-1
SD	20	22
Range	-83, 47	-75, 47
95%CI	-6, 3	-10, 0
P value	0.604	0.068
HDL-C		
Mean change	2	1
Median	1	1
SD	7	7
Range	-12, 22	-19, 20
95%CI	0, 3	-1, 2
P-value	0.022*	0.320

Abbreviations: TC, total cholesterol; TG, triglyceride; LDL-C, low density lipoprotein cholesterol; HDL-C, high density lipoprotein cholesterol; SD, standard deviation; CI, confidence interval. *P <0.05. Paired *t*-test was conducted.

Table S14. The mean change from week 0 to week 20 in exploratory laboratory parameters

	week	TENS<br>N = 78	Placebo<br>N = 77	*P* value
CRP (mg/dL)	0	0.22 (0.30)	0.24 (0.07)	0.095
	20	0.16 (0.23)	0.38 (1.12)	
Adiponectin	0	5.71 (4.61)	4.92 (4.64)	0.886
(μg/mL)	20	5.86 (4.79)	5.13 (4.05)	
TNF-α (pg/mL)	0	0.88 (0.48)	0.88 (0.71)	0.454
	20	0.86 (0.43)	0.90 (0.44)	
FGF-21 (ng/mL)	0	0.28 (0.25)	0.35 (0.33)	0.752
	20	0.24 (0.17)	0.32 (0.30)	

Abbreviations: CRP, C-reactive protein; TNF-α, tumor necrosis factor-α; FGF-21, fibroblast growth factor-21. Two-sample *t*-test was conducted for continuous variable. Data are presented as means (SD).

Differences within the group of CRP mean change from baseline in ITT population

	week	TENS<br>N = 78	Placebo<br>N = 77
Change from baseline	20-0	-0.06 (0.02)	0.13 (0.03)
P value		0.022*	0.848

Abbreviation: CRP, C-reactive protein. *Statistically significant; Wilcoxon signed rank test was conducted. Data are presented as means (SD).

Differences within the group of CRP mean change from baseline in PP population

	week	TENS<br>N = 74	Placebo<br>N = 73
Change from baseline	20-0	-0.06 (0.01)	0.13 (0.003)
P value		0.046*	0.848

Abbreviation: CRP, C-reactive protein. *Statistically significant; Wilcoxon signed rank test was conducted. Data are presented as means (SD).

Table S15. Full listing of adverse events

Summary of AEs by System Organ Class and Preferred Term by subject (Safety population)

Characteristic	TENS N=81	Placebo N=79	P-value
No. of patients with AEs	25 (30.86%)	28 (35.44%)	>0.999
No. of AEs	54	46	
No. of Patients with SAEs	3 (3.70%)	2 (2.53%)	0.744
No. of SAEs	4	5	
No. of patients with SADEs	0	0	NA
No. of SADEs	0	0	
No. of patients with UADEs	1 (1.23%)	1 (1.27%)	>0.999
No. of UADEs	3	3	
AE by severity (Patients)			0.213
Mild	19 (76.00%)	23 (82.14%)	
Moderate	6 (24.00%)	3 (10.71%)	
Severe	0	2 (7.14%)	
AE by severity (Events)			0.023*
Mild	45 (83.33%)	37 (80.43%)	
Moderate	9 (16.67%)	4 (8.70%)	
Severe	0	5 (10.87%)	
AE by relationship (Patients)			0.514
Certain	1 (4.00%)	0	
Probable/ Likely	1 (4.00%)	2 (7.14%)	
Possible	5 (20.00%)	5 (17.86%)	
Unlikely	3 (12.00%)	8 (28.57%)	
Not related	15 (60.00%)	13 (46.43%)	
AE by relationship (Events)			0.131
Certain	3 (5.56%)	0	
Probable/ Likely	1 (1.85%)	3 (6.52%)	
Possible	6 (11.11%)	11 (23.91%)	
Unlikely	13 (24.07%)	12 (26.09%)	
Not related	31 (57.41%)	20 (43.48%)	
AE by action taken to study device			0.330
None	50 (92.59%)	39 84.78%)	
Study device interrupted	0	1 (2.17%)	
Study device discontinued	4 (7.41%)	6 (13.04%)	

Characteristic	TENS<br>N=81	Placebo<br>N=79	P-value
AE by action taken- Other			0.001*
None	21 (38.89%)	33 (71.74%)	
Non-study treatment given	33 (61.11%)	13 (28.26%)	
AE by outcome			0.098
Completely recovered	28 (51.85%)	30 (65.22%)	
Recovered, residual effect	5 (9.26%)	5 (10.87%)	
Continuing	15 (27.78%)	11 (23.91%)	
Lost to follow-up	6 (11.11%)	0	

Abbreviations: AE: Adverse Event; SAE: Serious Adverse Event; SADE: Serious Adverse Device Effect; UADE: Unanticipated Adverse Device Effect. 'Severity', 'Relationship': Calculated based on the highest level of severity of each patient. Percentage of Action taken for study device/other = Number of events/Total AEs. Percentage of Outcome = Number of events/Total AEs. *Statistically significant. Mild: Transient, needed no special treatment, and/or did not interfere with the subject's daily activity; Moderate: Low level of inconvenience or concern to the subject and may interfere with daily activities, but was usually by simple therapeutic remedy; Severe: Interrupted a subject's daily activity and typically required intervening treatment.

Summary of AE related to study device by System Organ Class and Preferred Terms by subject (Safety population)

SOC<br>PT	TENS<br>N=81	Placebo<br>N=79
No. of patients with AEs	25 (30.86%)	28 (35.44%)
No. of AEs	54	46
No. of patients with related to study device	7 (8.64%)	7 (8.86%)
No. of AEs related to study device	10	14
Cardiac disorders	0	1
Palpitations	0	1
Metabolism and nutrition disorders	4 (4.94%)	6 (7.59%)
Hypoglycemia	3 (3.70%)	6 (7.59%)
Pseudohypoglycemia	0	1 (1.27%)
Investigations	1 (1.23%)	0
Blood creatine phosphokinase increased	1 (1.23%)	0

SOC<br>PT	TENS<br>N=81	Placebo<br>N=79
Nervous system disorders	0	1 (1.27%)
Dizziness	0	1 (1.27%)
General disorders and administration site conditions	2 (2.47%)	0
Pain	1 (1.23%)	0
Swelling	1 (1.23%)	0
Reproductive system and breast disorders	1 (1.23%)	0
Postmenopausal hemorrhage	1 (1.23%)	0
Skin and subcutaneous tissue disorders	1 (1.23%)	0
Erythema	1	0
Vascular disorders	1 (1.23%)	0
Hypotension	1	0

Abbreviations: AE, adverse events; SAE, severe adverse events; SOC, system organ class; PT, preferred terms. Percentage=Number of patients with events/Total subjects.

Summary of SAEs (Safety population)

Characteristic	TENS<br>N=81	Placebo<br>N=79	Total<br>N=160
No. of patients with AEs	25 (30.86%)	28 (35.44%)	53(33.12%)
No. of patients with SAEs	3 (3.70%)	2 (2.53%)	5 (3.12%)
No. of SAEs	4	5	9
No. of SAEs (excluded death)	4	5	9
No. of patients with SAEs (excluded death)	3 (3.70%)	2 (2.53%)	5 (3.12%)
No. of patients discontinued due to SAEs	0	1 (1.26%)	1 (0.62%)
No. of SAEs caused discontinuation	0	4	4

Abbreviations: AE, adverse event; SAE, serious adverse event

Characteristic	TENS<br>N=81	Placebo<br>N=79	Total<br>N=160

Number of subjects discontinued due to SAEs by system organ class and preferred term

SOC<br>PT	Total
No. of patients discontinued	6
No. of patients discontinued due to SAEs	1
No. of SAEs	9
Gastrointestinal disorders	1
Gastric ulcer	1
Hepatobiliary disorders	1
Bile duct stenosis	1
Hepatitis	1
Neoplasms benign, malignant and unspecified (incl cysts and polys)	1
Pancreatic carcinoma	1

SAE, serious adverse event

List of UADEs

Group	AE (PT)	Relationship	Severity	Outcome
TENS	Pain	Certain	Mild	Completely recovered
	Swelling	Certain	Mild	Completely recovered
	Erythema	Certain	Mild	Completely recovered
Placebo	Hypoglycemia	Possible	Mild	Completely recovered
	Hypoglycemia	Possible	Mild	Completely recovered
	Pseudo-hypoglycemia	Possible	Mild	Completely recovered

Abbreviations: UADE, unanticipated adverse device effect; AE, adverse event; PT, preferred term. Mild: Transient, needed no special treatment, and/or did not interfere with the subject's

daily activity; Moderate: Low level of inconvenience or concern to the subject and may interfere with daily activities, but was usually by simple therapeutic remedy; Severe: Interrupted a subject's daily activity and typically required intervening treatment.

Table S16. Summary of Insulin concentrations by Visit (Safety Population)

Insulin, mU/L	TENS		Placebo
Visit 2, week 0			
N	81		79
Mean	11.38		10.17
SD	8.14		6.4
Median	8.8		8.1
Range (min, max)	1.3, 40.7		2.6, 31.5
95% CI	9.6, 13.2		8.7, 11.6
P value		0.298	
Visit 4, week 4			
N	81		77
Mean	11.64		9.62
SD	10.63		7.02
Median	8.6		8.1
Range (min, max)	1.5, 79.8		1.5, 35.8
95% CI	9.3, 14		8.0, 11.2
P value		0.160	
Visit 6, week 16			
N	78		76
Mean	10.01		9.76
SD	6.74		6.19
Median	8.40		8.30
Range (min, max)	1.0, 39.0		2.3, 37.8
95% CI	8.5, 11.5		8.3, 11.2
P value		0.812	
Visit 8, week 20			
N	79		75
Mean	9.90		9.37
SD	8.02		5.90
Median	8.50		8.30
Range (min, max)	1.0, 61.6		2.3, 28.6
95% CI	8.1, 11.7		8.0, 10.7
P value		0.636	

Abbreviations: SD, standard deviation; CI, confidence interval. Two sample t-test was conducted. If normal assumption was violated, Wilcoxon rank sum test would be applied. *Statistically significant.

Figures

Supplementary Fig. S1. Schematic design of the study.

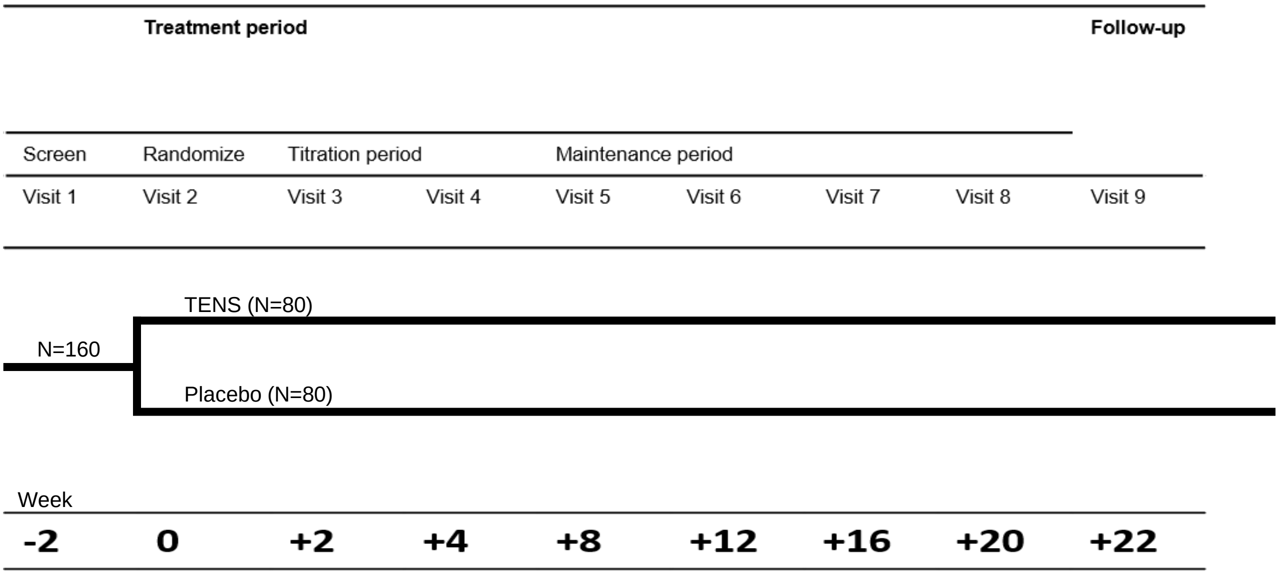

TENS, Transcutaneous Electrical Nerve Stimulator.

Supplementary Fig. S2. Subject disposition flowchart of each treatment group. ITT, intention-to-treat; PP, per-protocol.

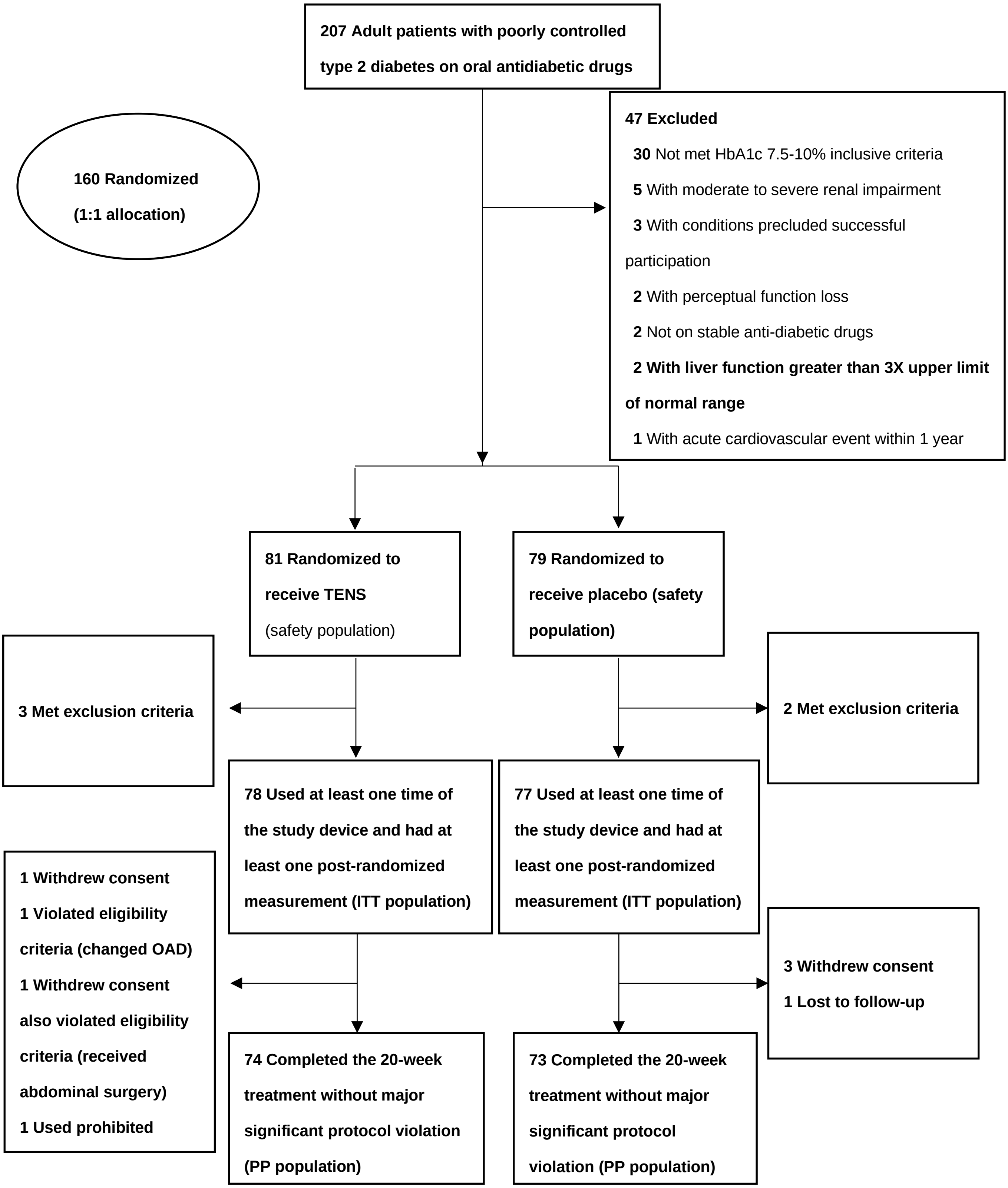

Supplementary Fig. S3. Homeostasis model assessment of **(A)** β cell function (HOMA-β) and **(B)** insulin resistance (HOMA-IR) from week 0 to week 20 in the Transcutaneous Electrical Nerve Stimulator (TENS) vs. the placebo group in safety population were analyzed applying the formulas as reported before[1]. Mixed model two–way ANOVA and two-sample *t*-test were carried out and presented with means ± standard error of mean (SEM), ns, non-significant.

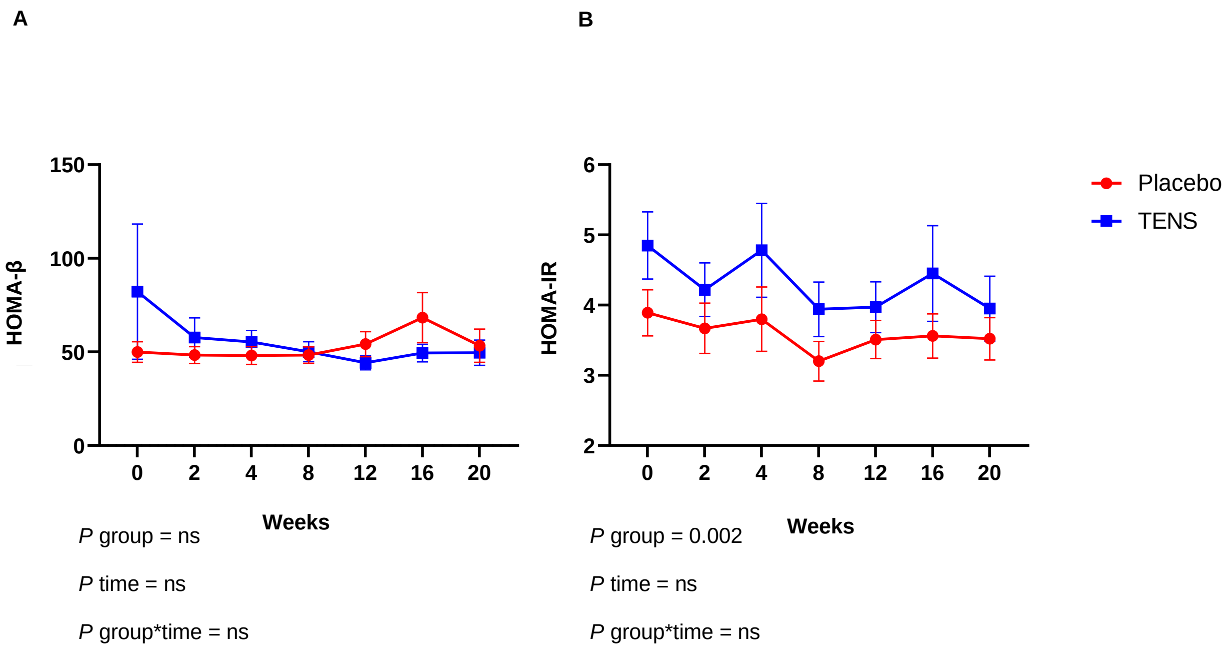

Reference

1. Onishi Y, Hayashi T, Sato KK, et al. Fasting tests of insulin secretion and sensitivity predict future prediabetes in Japanese with normal glucose tolerance. J Diabetes Investig 2010;1(5):191-5. DOI: 10.1111/j.2040-1124.2010.00041.x.

eAppendix

DW1330 Information

The study device, "Dragon Waves Resonant Home Care" Electronic Nerve Stimulator (DW1330), indicated to relieve pain, reduce the sensitivity of peripheral nerve system, and stimulate blood circulation, has been approved for marketing in Taiwan. The impulses are sent through wires to patches which are placed at appropriate body sites. DW1330 uses full-frequency wave resonant technology while the principle is similar to other electrical stimulation devices, such as transcutaneous electrical nerve stimulation, low frequency therapeutic device, or middle frequency stimulator, but it is applied with different frequency between 1 and 100 Hertz (HZ). During the stimulation period, DW1330 will deliver predefined different mixed frequency. DW1330 is a small portable, battery-operated device along with wires and 2 patches. The blueprint of DW1330 and the design of mixed frequency are shown in **Figure 1** and **Figure 2**. The model of electronic stimulation is shown in **Figure 3**.

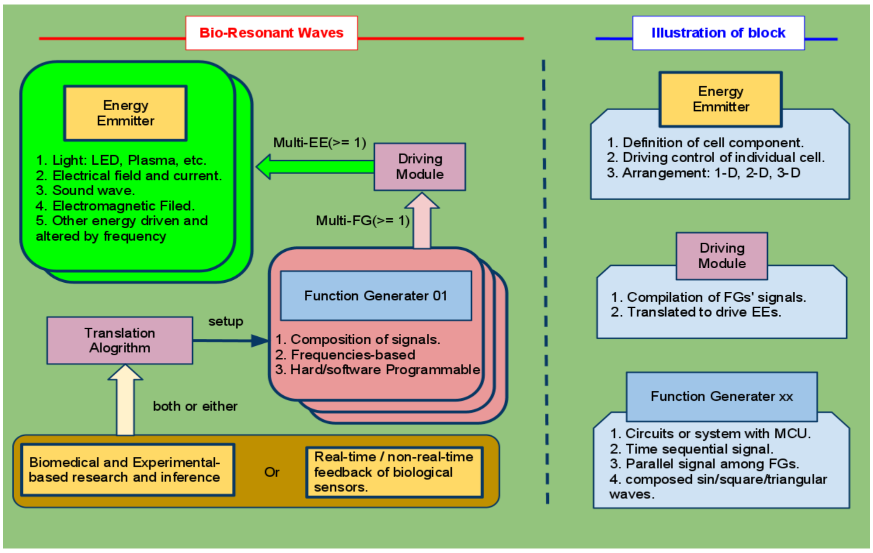

Figure 1 Blueprint of DW1330

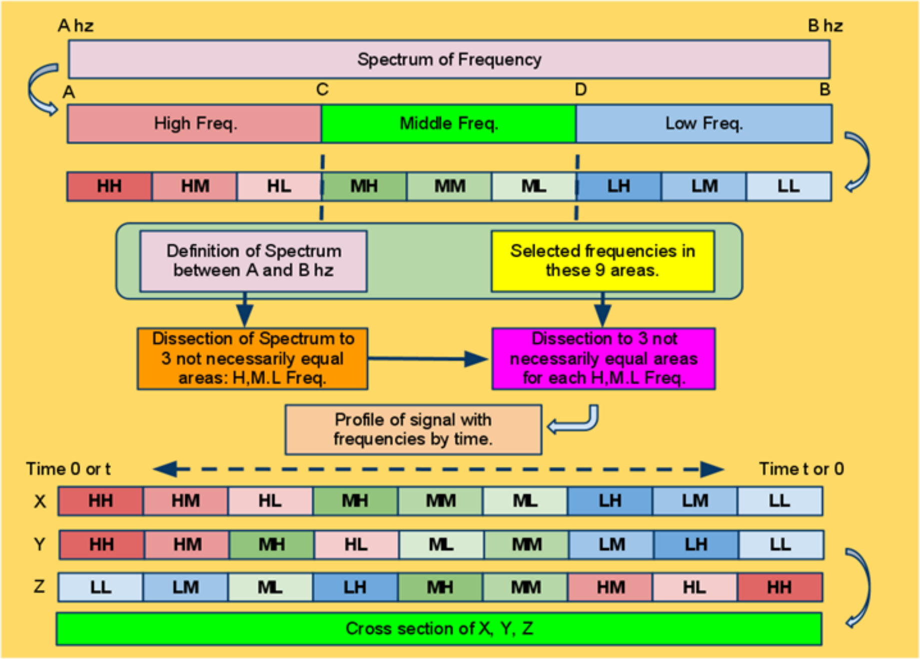

Figure 2 Set up of DW1330 frequency.

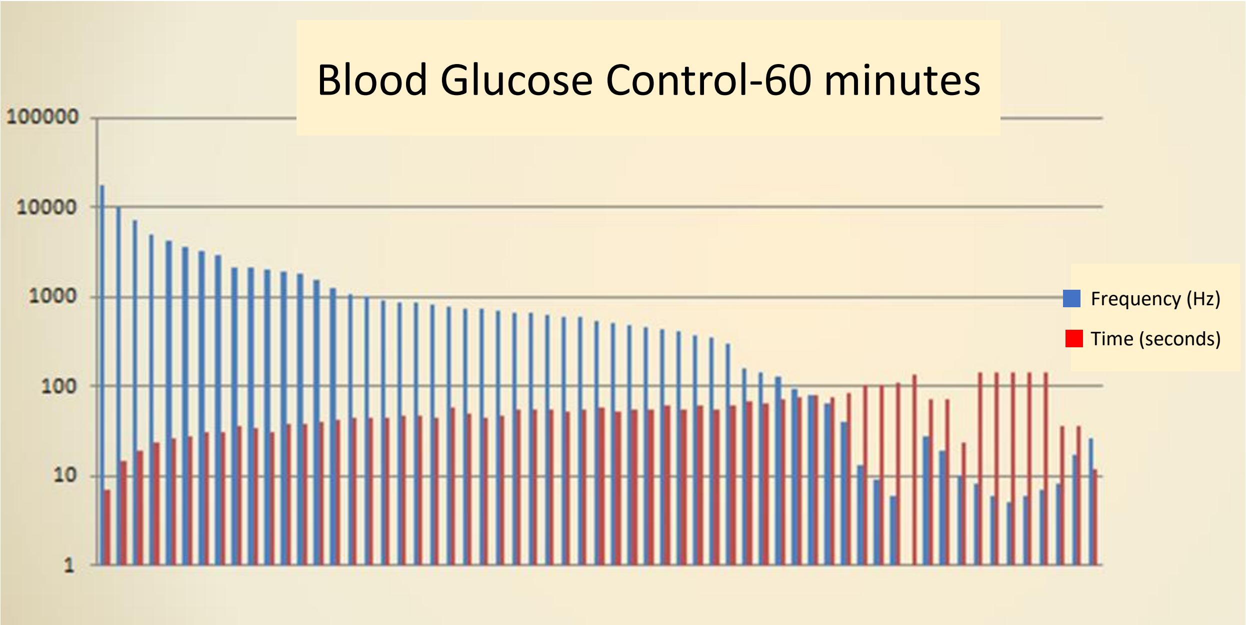

Figure 3 The electronic stimulation model of DW1330.

Animal Study

In pre-clinical setting, DW1330 has been proven to significantly improve glucose regulation in animal model, as indicated by fasting plasma glucose (FPG) and HbA1c (refer to **Figure 4** and **Figure 5**). The study is reported in accordance with ARRIVE guidelines

(https://arriveguidelines.org). After a 6-week treatment period, FPG did not differ between the healthy mice without resonant wave treatment, healthy mice with resonant wave treatment, and diabetic mice receiving resonant wave treatment, but it achieved statistically significant difference between the diabetic mice with and without resonant wave treatment. As for HbA1c, the value was much lower in diabetic mice treated with resonant wave as compared with those without resonant wave treatment (5.4% in diabetic mice with resonant wave treatment versus 7.6% in diabetic mice without resonant wave treatment). The effects of resonant wave treatments on vital organs were evaluated after the mice were sacrificed by carbon dioxide (CO_2) inhalation, and results showed that resonant wave intervention reversed the pathological changes on liver, kidney, pancreas, epididymal adipose tissues, and lung in mice with diabetes mellitus induced by streptozotocin. The results indicated that DW1330 could reduce FPG level and HbA1c and was safe in animal model.

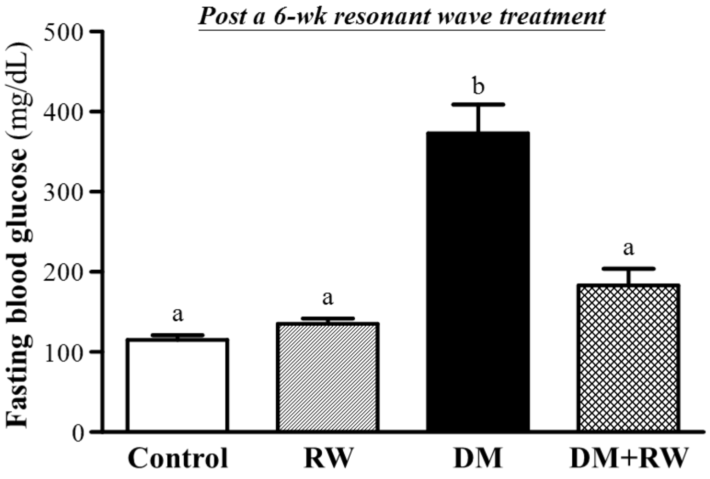

Figure 4 Fasting blood glucose after a 6-week treatment.

Control: health mice without resonant wave treatment, RW: healthy mice with resonant wave treatment, DM: diabetic mice without resonant wave treatment, DM+RW: diabetic mice with resonant treatment.

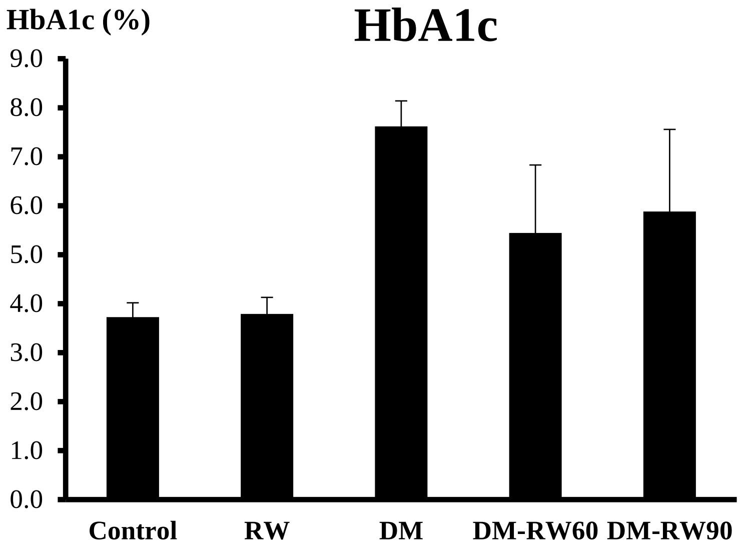

Figure 5 Fasting blood glucose after a 6-week treatment.

Control: healthy mice without resonant wave treatment, RW: health mice with resonant wave treatment, DM: diabetic mice without resonant wave treatment, DM-RW60: diabetic mice with 60 minutes of resonant treatment daily, DM-RW90: diabetic mice with 90 minutes of resonant treatment daily.

Human Study

In an unpublished study conducted in Taiwan, subjects were randomized to either receive DW1330 or placebo for 12 weeks and then crossed over to the alternate arm for 12 weeks. Subjects were instructed to use the device (with effective or ineffective frequency) for 60 minutes daily for 5 days per week during the study period. The results showed that DW1330 significantly improved glycemic control in T2DM patients, as indicated by HbA1c. The HbA1c level decreased by approximately 0.5% in patients with higher body mass index (≥26 kg/m^2) after a 12-week treatment with DW1330.

Subjects received their study device and applicable training and were instructed to use the device 1 hour after dinner by attaching the patches to the left and right side, around 5-10 cm from the navel as shown in **Figure 6**. The frequency of device use was one hour per day, 5 days/week.

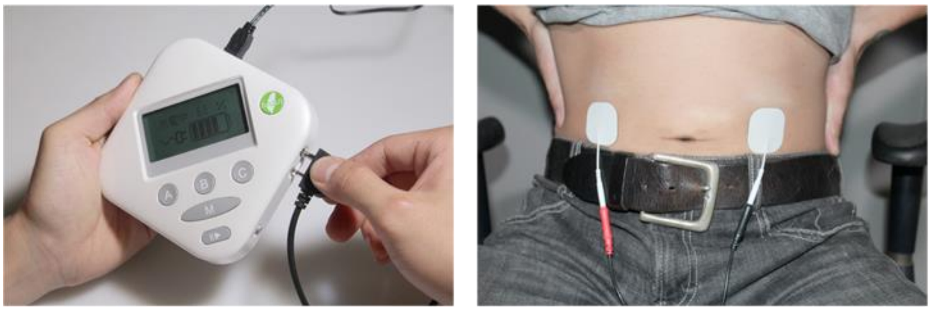

Figure 6 Instrumentation schematic. Left: the user interface of DW1330, the parameter A, B, and C are all the same electronic model setting; Right: the location of DW1330 patches attaching.
